# Targeted Screening Studies on Endemic Propolis Extracts for Selective Cytotoxicity Against Human Melanoma Cells Models

**DOI:** 10.3390/molecules31213841

**Published:** 2026-10-24

**Authors:** Marta Trzaskowska, Jarosław Widelski, Piotr Okińczyc, Paulina Kazimierczak, Vladyslav Vivcharenko, Anna Bozhadze, Malkhaz Jokhadze, Agata Przekora

**Affiliations:** 1 Institute of Pharmaceutical Sciences, Faculty of Medicine, KUL-The John Paul II Catholic University of Lublin, Konstantynów 1 H Street, 20-708 Lublin, Poland; marta.trzaskowska@kul.pl (M.T.); paulina.kazimierczak@kul.pl (P.K.); vladyslav.vivcharenko@kul.pl (V.V.); 2 Department of Tissue Engineering and Regenerative Medicine, Medical University of Lublin, Chodzki 1 Street, 20-093 Lublin, Poland; 3 Department of Pharmacognosy with Medicinal Plants Garden, Medical University of Lublin, Chodzki 1 Street, 20-093 Lublin, Poland; jaroslaw.widelski@umlub.pl; 4 Department of Pharmacognosy and Herbal Medicines, Wroclaw Medical University, Borowska 211a Street, 50-556 Wrocław, Poland; piotr.okinczyc@umw.edu.pl; 5 Department of Pharmacognosy, Tbilisi State Medical University, 33 Vazha-Pshavela Ave, 0186 Tbilisi, Georgia; a.bozhadze@tsmu.edu; 6 Department of Pharmaceutical Botany, Tbilisi State Medical University, 33 Vazha-Pshavela Ave, 0186 Tbilisi, Georgia; m.jokhadze@tsmu.edu

**Keywords:** cytotoxicity, antioxidant activity, polyphenols, anticancer activity, skin cancer, apoptosis

## Abstract

Melanoma is the most aggressive and life-threatening form of skin cancer. Propolis possesses diverse biological activities and is considered a promising source of compounds for anticancer therapy. This study evaluated the cytotoxic effects of propolis from endemic regions of Nepal, Greece, Kazakhstan, Ukraine, and Georgia on melanoma cell lines (A375, SK-MEL-3) and normal human skin fibroblasts (BJ). Chemical characterization was performed using UHPLC-UV-ESI-qTOF-MS/MS. Total flavonoid and polyphenol contents, as well as antioxidant activity, were also determined. Biological effects were assessed by measuring cell viability, apoptosis (fluorescent staining and confocal microscopy), and caspase-6 activity (fluorometric assay). Most analyzed extracts contained markers characteristic of black poplar-type propolis, including chrysin, pinocembrin, galangin, pinobanksin-3-acetate, and caffeic acid monoesters such as caffeic acid phenethyl ester (CAPE) and isomers of the 1,1-dimethylallyl ester. Three propolis samples from Georgia showed the strongest anticancer activity against melanoma cells while exhibiting low toxicity toward normal fibroblasts (SI > 1). These extracts also displayed high antioxidant activity and elevated polyphenol content. Apoptosis predominated in A375 cells, whereas necrosis was more evident in SK-MEL-3 cells, consistent with increased caspase-6 activity in A375 cells. These results suggest that Georgian propolis may be a promising candidate for further investigation in melanoma therapy.

## 1. Introduction

Melanoma is the most lethal form of skin cancer, caused by the abnormal proliferation of melanocytes (neural crest-derived cells) in the basal layer of the epidermis, which produce melanin (a UV-absorbing polymer) [1]. The incidence of melanoma has risen markedly over the past decades. In the United States, incidence rates increased from 7.9 to 25.3 per 100,000 population between 1975 and 2018, corresponding to an overall increase of more than 320%. [2]. Incidence also varies significantly by region. The highest incidence rate was recorded in Australia and New Zealand (35.8), and the lowest in Africa and Asia (East Asia = 0.39, North Africa = 0.45). However, in Western European countries, the incidence was 18.9, and in Northern Europe, it was 17.8 [3,4]. Despite representing only 1% of skin cancers, melanoma is responsible for over 80% of skin cancer-related deaths. Risk factors for melanoma include sun exposure, indoor tanning, fair skin, family history of skin cancer, a high number of nevi/moles, and immunodeficiency [1,2,5]. Surgical excision with extended margins is the primary treatment of localized melanomas, followed by radiotherapy, cryotherapy, and chemotherapy, which can be used alone or in combination [1]. However, there remains a high risk of local recurrence in skin cancer therapy [6]. Consequently, chemopreventive interventions may be an effective strategy to prevent or delay carcinogenesis. Various chemopreventive agents include vitamins, statins, natural products (e.g., resveratrol, curcumin, green tea polyphenols), or non-steroidal anti-inflammatory drugs [7].

For years, animal-derived products such as honey and propolis have been used in traditional natural therapies and folk medicine to treat skin lesions. Propolis (also known as bee glue) is a brownish, resinous product made by honeybees, mainly *Apis mellifera* L., and has been utilized as a source of bioactive agents [8]. A substantial amount of evidence indicates that propolis exhibits a broad spectrum of biological and therapeutic activities, which are largely attributed to its diverse composition of phenolic acids, flavonoids, terpenoids, and other bioactive constituents. The antioxidant activity of propolis represents an extensively investigated biological property. Its phenolic and flavonoid constituents can act as free-radical scavengers and may contribute to the neutralization of reactive oxygen and nitrogen species [9]. This antioxidant potential may also contribute to several of its other biological effects, including its anti-inflammatory and antitumor activities. The immunomodulatory properties of propolis have been associated with its ability to modulate the production of cytokines and other inflammatory mediators. Its anti-inflammatory activity has been demonstrated in different experimental models and may be mediated by the inhibition of pro-inflammatory signaling pathways, including nitric oxide (NO) production in activated macrophages and the synthesis of prostaglandin E2 (PGE2), thromboxane A2, and interleukin IL-1β [9,10]. Particularly noteworthy is also the antitumor and anticancer potential of propolis and its individual constituents. Experimental studies have demonstrated inhibitory effects against various cancer cell types, including human fibrosarcoma, lung adenocarcinoma, renal cell carcinoma, colorectal cancer cells and melanoma [10,11]. Propolis also exhibits broad-spectrum antimicrobial activity. Its antibacterial effects have been reported against both Gram-positive and Gram-negative bacteria, including *Staphylococcus aureus*, *Mycobactrium tuberculosis* and *Klebsiella pneumoniae* [9,12]. Similarly, antifungal activity has been demonstrated against several pathogenic and opportunistic fungi, including *Aspergillus niger*, *Aspergillus flavus*, and *Candida albicans* [12,13]. In addition, propolis has been reported to exhibit antiprotozoal and antiviral properties [12], further highlighting its potential as a source of compounds with activity against a broad range of microorganisms. Beyond its antimicrobial and anticancer properties, propolis has been investigated for a range of additional biological effects. These include local anesthetic activity, hepatoprotective, antimutagenic [12] and anti-genotoxic effects [14], and potential neuroprotective and antineurodegenerative properties [8,10,11,15,16,17,18]. The healing mechanism of propolis remains a subject of debate, but it is theorized that its effects are likely due to the synergistic interactions among its chemical constituents [8]. Regarding skin wound healing, propolis can enhance reepithelialization, promote fibroblast growth factor-2 expression, boost collagen synthesis, and stimulate keratinocyte and fibroblast proliferation, contributing to more efficient and faster skin regeneration [19]. On the other hand, the activity of propolis against melanoma is still not well-understood. However, studies have shown that propolis can inhibit specific oncogenic signaling pathways (e.g., β-catenin, NF-κB, c-myc, PI3K/AKT), leading to reduced cancer cell proliferation and growth [10]. It was also proven that baccharin and *p*-coumaric acid extracted from Brazilian green propolis decreased the number of inflammatory cells recruited to the tumor region in melanoma-bearing mice [20].

Furthermore, many types of propolis exhibit high tyrosinase inhibition activity [21]. Tyrosinase is a key enzyme in the synthesis of melanin, the skin pigment. Increased melanin synthesis due to tyrosinase hyperactivity (triggered by ageing and skin inflammation) can cause hyperpigmentation disorders. The role of melanin, particularly in terms of its overproduction, in the development of malignant melanoma—a dangerous type of cancer that develops in melanocytes—is of great importance in terms of prevention. Abnormal melanogenesis can be prevented and controlled by inhibiting tyrosinase activity [21,22,23,24,25].

Propolis is characterized by a complex composition, consisting of resin and balsam (50%), wax (30%), pollen (5%), essential and aromatic oils (10%), and other substances such as organic debris (5%). Its chemical composition plays a crucial role in its pharmacological activities and includes phenolic acids, flavonoids, diterpenes, sesquiterpenes, esters, lignans, alcohols, aromatic aldehydes, amino acids, fatty acids, vitamins, and minerals [8,10]. It is important to note that the chemical composition of propolis is highly variable, depending on geographical origin. Observed differences may additionally be influenced by the period of collection (e.g., month), prevailing climatic conditions, seasonal variation, and beekeeping practices. Notably, propolis samples originating from the same country have been shown to differ in both chemical composition and biological activity. Furthermore, some studies indicate that the method of collection may exert a greater effect on propolis quality than the geographical region of origin. Research has shown that the composition of propolis, the individual concentrations of components, and the antibacterial activity of propolis may also vary depending on the races of bee that collected them—despite the fact that the propolis was obtained from the same region and the bees came from the same apiary [11,26,27]. The aim of this screening study was to comprehensively evaluate the biomedical potential of propolis derived from endemic areas of Nepal, Greece, Kazakhstan, Ukraine, and Georgia to identify the propolis with the highest anti-melanoma activity. The chemical composition, antioxidant activity, and cytotoxicity of propolis extracts against melanoma cells and normal skin cells were assessed.

## 2. Results and Discussion

### 2.1. Chemical Composition of Propolis Extracts

Propolis constitutes a highly heterogeneous natural matrix comprising a wide spectrum of chemically diverse constituents. In samples of mixed botanical provenance, the principal analytical difficulty stems from the co-occurrence of structurally related compounds—particularly phenolic acid glycerides, monoesters, and selected flavonoid aglycones—which display comparable retention behavior and similar interactions with chromatographic stationary phases, thereby complicating their efficient resolution.

**Figure 1:**
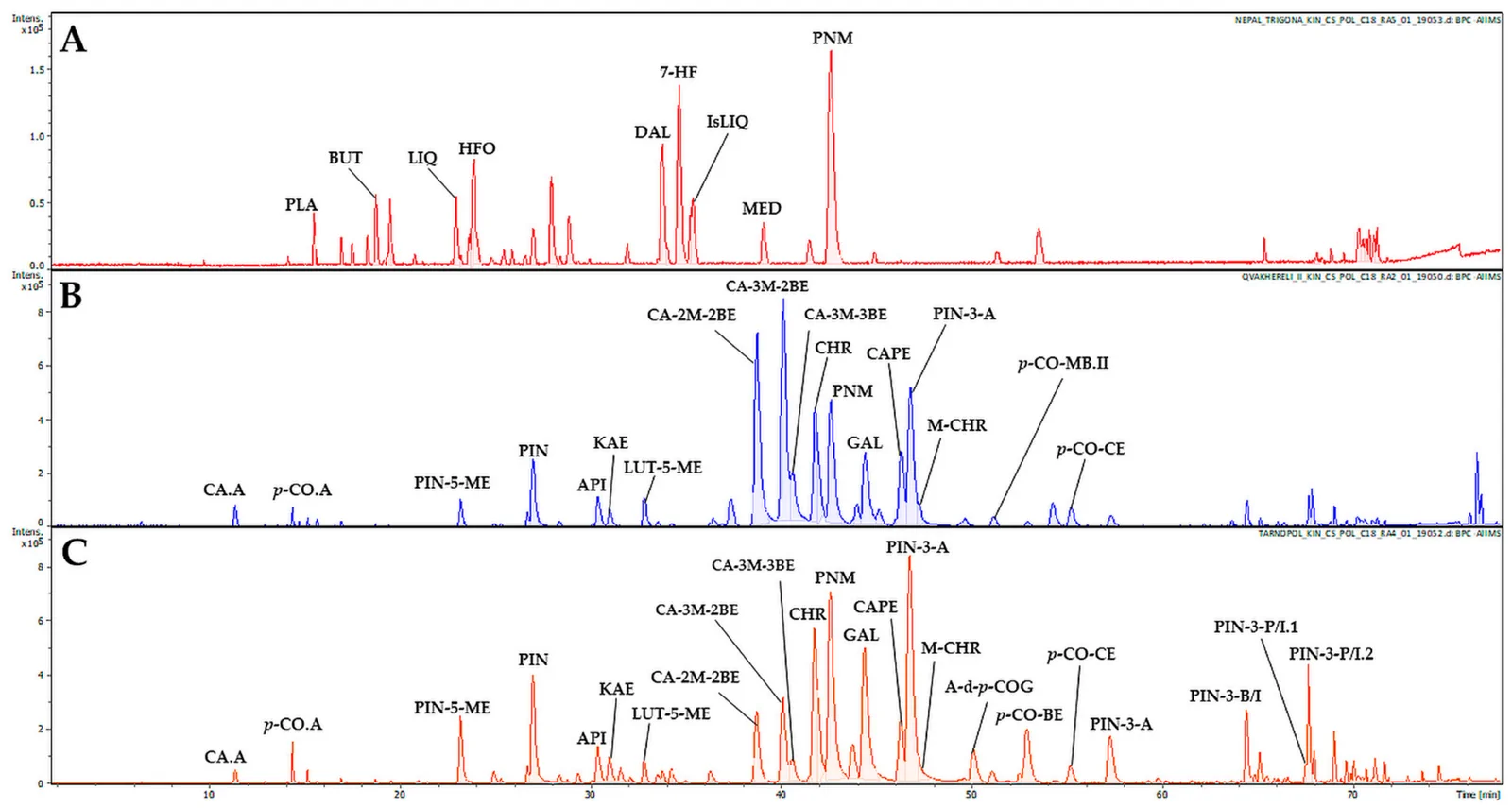
Comparison of UHPLC-MS basic peak chromatograms of representative propolis samples in electrospray negative mode. Figure legend: Chromatograms of propolis extracts: (**A**)—Nepalese propolis (NE-TRI); (**B**)—Georgia, Qvakhreli sample 2 (GE-Q2); (**C**)—Ukraine, Tarnopol (UK-TA). Component abbreviations: CA.A—caffeic acid; *p*-CO.A—*p*-coumaric acid; PLA—plathymenin; BUT—butin; LIQ—liquiritigenin; PIN-5-ME—pinobanksin-5-methyl ether; HFO—2′-hydroxyformononetin; PIN—pinobanksin; API—apigenin; KAE—kaempferol; LUT-5-ME—luteolin-5-methyl ether; DAL—dalbergin; 7-HF—7-hydroxyflavanone; IsLIQ—isoliquiritigenin; CA-2M-2BE—caffeic acid 2-methyl-2-butenyl ester; MED—medicarpin; CA-3M-2BE—caffeic acid 3-methyl-2-butenyl ester; CA-3M-3BE—caffeic acid 3-methyl-3-butenyl ester; CHR—chrysin; PNM—pinocembrin; GAL—galangin; CAPE—caffeic acid phenethyl ester; PIN-3-A—pinobanksin-3-acetate; M-CHR—methoxychrysin; A-d-p-COG—2-acetyl-1,3-di-*p*-coumaroylglycerol; *p*-CO-MB.II—*p*-coumaric acid 3-methyl-2-butenyl or 2-methyl-2-butenyl; *p*-CO-BE—*p*-coumaric acid benzyl ester; *p*-CO-CE—*p*-coumaric acid cinnamyl ester; PIN-3-A—pinobanksin-3-propanoate; PIN-3-B/I—pinobanksin-3-butanoate or isobutanoate; PIN-3-P/I.1—pinobanksin-3-pentanoate or isopentanoate isomer I; PIN-3-P/I.2—pinobanksin-3-pentanoate or isopentanoate isomer II.

Within this study, propolis extracts from Nepal, Greece, Kazakhstan, Ukraine, and Georgia were tested (Table 1). Results of the phytochemical analysis are presented in Figure 1, Supplementary Materials Tables S1 and S2. Identification procedures of components in several propolis extracts were described in our previous research (Nepal—NE-TRI [28], Greece—GR-PA, Ukraine—UK-TA [26], Georgia—region Qvakhvreli sample 1—GE-Q1, Georgia region Ota—GE-OT; Georgia—region Orgora—GE-OR [29], Kazakhstan—region Bozovoe—KA-BO [30]). However, some propolis samples were investigated within this study for the first time (Georgia—region Qvakhvreli sample 2—GE-Q2, Georgia—region Vasisubani sample 1—GE-VA1, Georgia—region Vasisubani sample 2—GE-VA2), while others (NE-TRI, GR-PA, UK-TA) were re-examined using UHPLC-UV-ESI-qTOF-MS/MS, a method different from that used in the previously published article. This approach enabled the determination of the phytochemical profiles of all investigated propolis samples using the same analytical technique (UHPLC-UV-ESI-qTOF-MS/MS), allowing a reliable comparison of the obtained data.

UHPLC-DAD-MS/MS analysis, based on separation using Kinetex^®^ core-shell Polar C18, enabled the detection of 78 compounds in the ethanolic extracts of propolis samples (GE-VA1, GE-VA2 and GE-Q2) studied for the first time.

The samples of Georgian propolis contained characteristic compound groups that are typical of this product such as flavonoids and their derivatives (chrysin, pinocembrin, pinocembrin-3-acetate, apigenin, kaempferol, pinobanksin-5-methyl ether, luteolin-5-methyl ether), as well as free phenolic acids (caffeic acid, *p*-coumaric acid, and their esters (caffeic acid 2-methyl-2-butenyl ester, caffeic acid 3-methyl-2-butenyl ester, *p*-coumaric acid cinnamyl ester, caffeic acid phenethyl ester—CAPE, *p*-coumaric acid 3-methyl-2-butenyl ester) (Supplementary Materials Table S2).

In general, most of propolis extracts (Georgian, Kazakhstani, Greek and Ukrainian) exhibited a strong presence of black poplar markers (chrysin, pinocembrin, galangin, pinobanksin-3-acetate as well as caffeic acids monoesters, especially caffeic acid phenethyl ester—CAPE and different isomers of 1,1-dimethylallyl ester) and low or trace markers of aspen propolis (phenolic acid glycerides, especially 2-acetyl-1,3-di-*p*-coumaroylglycerol). Presence of aspen markers was strongest in the GE-OR, but they were significantly weaker than black poplar markers (Supplementary Materials Table S2). For this reason, most of the samples should be classified as of the black poplar propolis type.

The Nepalese propolis exhibited a completely different phytochemical profile from other samples tested. It had some common substances, like pinobanksin, pinobanksin-5-methyl ether and pinocembrin, but it also contained pinocembrin at a higher relative amount than trace. Main components of Nepalese propolis included 2′-hydroxyformononetin, dalbergin, 7-hydroxyflavanone, pinocembrin and some unidentified constituents. Based on the obtained phytochemical profile, it was assumed that *Dalbergia* sp. plant was a precursor of Nepalese propolis.

### 2.2. Total Flavonoid, Total Phenolic Content and Antioxidant Potential

The analysis results showed that the total phenolic (TP) content ranged from 102.18 ± 1.31 (GE-VA2) to 286.77 ± 1.74 (GE-Q2) mgGAE/g extract (mg of gallic acid equivalents in g of dry extract) (Table 2).

The highest TP content was recorded in propolis extracts from Georgia: GE-Q2, GE-OT, GE-VA1, GR-PA, GE-Q1 (>218 mgGAE/g extract). The total flavonoid (TF) content ranged from 34.79 ± 1.71 (NE-TRI) to 219.69 ± 1.60 (GR-PA) mgQE/g extract (mg of quercetin equivalents in g of dry extract). The extracts with the highest TF content were samples from Greece and Ukraine (GR-PA and UK-TA). The results presented here are consistent with the available literature since comparable amounts of flavonoids and phenols were detected in materials collected in various European countries, including Georgia, and in Asian countries [29,31,32]. Most of the tested propolis samples were characterized by a higher content of polyphenols than flavonoids. The DPPH (2,2-diphenyl-1-picrylhydrazyl radical scavenging activity) test results for propolis extracts ranged from 20.94 ± 0.07 (GE-VA2) to 117.71 ± 1.04 (GE-OR) mgGAE/g (mg of gallic acid equivalents in g of dry extract). The strongest antioxidant activity in the DPPH assay was demonstrated by the GE-OR, GE-Q1, and GE-Q2 samples. The values obtained in the DPPH test were comparable to measurements reported by other authors in propolis samples from European countries, including Georgia [29,31]. FRAP analysis revealed values ranging from 6.82 ± 0.13 (NE-TRI) to 19.09 ± 0.04 (GE-Q2) mmol Fe^2+^/g extract (mmol Fe^2+^ equivalents in g dry extract). GE-Q1 and GA-Q2 propolis extracts demonstrated the highest antioxidant activity in the FRAP assay. It should be noted that the antioxidant activity of plant extracts is primarily due to the presence of flavonoids and polyphenols [33]. Statistical analyses showed strong correlation between TP and both antioxidant tests (DPPH and FRAP), while TF was not correlated (Table 3). Moreover, DPPH was also correlated with FRAP. This phenomenon may be explained by the higher activity of non-flavonoid polyphenols in DPPH and FRAP tests. These polyphenols were different hydroxycinnamic acids (*p*-coumaric, caffeic, ferulic and isoferluc—all water soluble) and their different esters. All mentioned components are well-known as strong radical scavengers, usually stronger than flavonoids. Antioxidant activity of the same flavonoids, especially relatively non-polar flavonoid aglycones, is connected with the direct protection of cell membranes and interaction with cell metabolism. To sum up, TF was not correlated with both DPPH and the FRAP test since DPPH evaluates direct radical scavenging activity, while FRAP requires water for the reaction.

It is worth noting that given the complex nature of antioxidant activity, it is beneficial to employ at least two complementary assays for its assessment. A single method cannot fully reflect the reactions occurring in vivo [34]. The DPPH test is based on the scavenging activity of stable free radicals, and the reaction mechanism involves the transfer of hydrogen atoms. The FRAP test, on the other hand, involves the reduction of metal ions (iron ion reduction) and the transfer of a single electron. Both tests have limitations, the FRAP assay is performed at a low, non-physiological pH, and DPPH has a different chemical structure compared to natural radicals [29,35]. However, both test procedures are facile, quick, and inexpensive to perform, making them useful for the preliminary determination of antioxidant properties in broad-based screening. Antioxidant compounds counteract the activity and overproduction of reactive oxygen species (ROS), and therefore oxidative stress that can lead to damage to macromolecules, including DNA [29,36].

### 2.3. Cytotoxicity Against Melanoma and Normal Skin Cells

**Figure 2:**
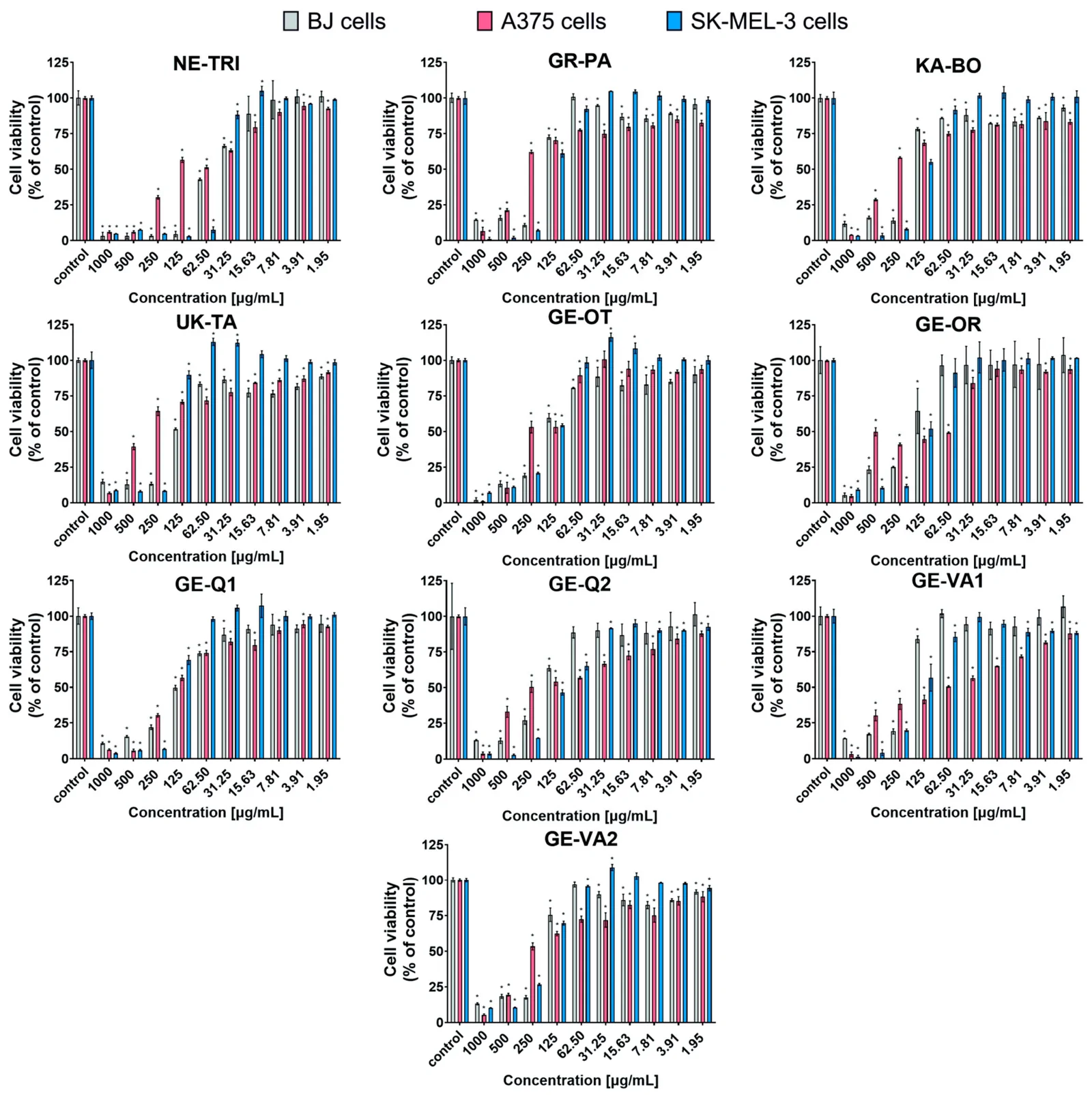
Cytotoxicity assessment of propolis extracts against normal skin fibroblasts (BJ cell line) and malignant melanoma cells (A375 and SK-MEL-3 cell lines) using the MTT assay. The test was performed after 24-h exposure to propolis extracts. Statistical analysis: *p* < 0.05, one-way ANOVA with Dunnett’s test; * indicates statistically significant differences compared with the corresponding control cells cultured in the growth medium without propolis extract.

The cytotoxicity of the propolis extracts was determined against two tumor cell lines of malignant melanoma: A375 and SK-MEL-3, as well as against normal skin cells: human fibroblasts (BJ). Propolis samples inhibited cell viability in a concentration- and cell line-dependent manner. A wide range of sample concentrations (1000–1.95 µg/mL) was tested in the experiment. Concentrations in the 1000–125 µg/mL range significantly reduced the viability of all tested cell lines, while the toxicity of concentrations ≤ 62.50 µg/mL was cell line-dependent (Figure 2). The tumor cell lines used in this study also differ in their sensitivity to the tested compounds. SK-ME-3 cells were more sensitive to the antitumor activity of most propolis samples (except GE-Q1, GE-VA1, and GE-VA2) compared to A375 cells. Potential anticancer agents should show safety index, also known as the selectivity index (SI) > 1, which indicates their selective cytotoxicity towards cancer cells. Thus, an anticancer agent revealing high SI (>1) is less cytotoxic against normal cells compared to the cancer cells [37]. Propolis extracts marked as NE-TRI, GR-PA, KA-BO, UK-TA, GE-OT, and GE-Q1 showed higher toxicity against normal BJ cells compared to A375 cells, as indicated by SI < 1 (Table 4). This trend was also demonstrated by comparing the IC_50_ concentrations of BJ and SK-MEL-3 cells for UK-TA, GE-OT, GE-Q1, and GE-VA2 propolis samples. NE-TRI propolis had the highest anticancer activity against SK-MEL-3 and very high towards A375, with the IC_50_ of 81.3 µg/mL for A375 and 42.6 µg/mL for SK-MEL-3. However, the SI of this propolis was low, especially for A375 cells (SI = 0.58), indicating high toxicity also towards normal skin cells—the lower the SI values estimated, the less safe the compound (i.e., more toxic towards normal cells). In contrast, the propolis extracts with the highest antimelanoma activity and selectivity, and therefore clinical potential, came from Georgia: GE-OR (SI = 1.19 for A375, SI = 1.34 for SK-MEL-3), GE-Q2 (SI = 1.42 for A375, SI = 1.61 for SK-MEL-3), and GE-VA1 (SI = 3.06 for A375, SI = 1.32 for SK-MEL-3). They demonstrated the highest cytotoxicity against melanoma cells of both lines while simultaneously exhibiting the lowest toxicity towards normal skin cells, as indicated by a high SI (>1). These propolis samples (GE-OR, GE-Q2, and GE-VA1) were selected for further analyses. The SI value of GE-VA1 propolis for A375 cells was particularly noteworthy, reaching 3.06.

The selected melanoma cell lines for these studies differ in their genetic profiles, origin, and melanin production. The A375 cell line contains BRAF V600E and CDKN2A mutations and was derived from a primary malignant melanoma, whereas SK-MEL-3 carries BRAF V600E and TP53 mutations and originated from a lymph node metastasis. Importantly, the two cell lines differ in their melanin-producing capacity: A375 is an amelanotic melanoma cell line, while SK-MEL-3 produces melanin [38,39]. Literature data indicate that the response of melanoma cells to therapeutic agents may vary significantly depending on their degree of pigmentation. These differences were observed both between amelanotic and melanotic cell lines, as well as between individual melanotic cell lines [38]. For example, Rok et al. showed that tetracyclines were characterized by approximately 20-fold higher EC_50_ values against amelanotic melanoma cells (A375 and C32) compared to melanotic COLO829 cells [40]. Bratu et al., in turn, reported a greater sensitivity of non-pigmented SK-MEL-5 cells to betulinic acid compared to pigmented B16-F10 melanoma cells [41]. Pinho et al. demonstrated that a hybrid molecule (HM, containing a triazene fragment linked to a sulfur L-tyrosine analogue) showed lower IC_50_ values against amelanotic A375 cells than against the melanotic MNT-1 cell line (38.7 µM and > 75 µM, respectively) [38]. Researchers suggested that the level of pigmentation of melanoma cells may be one factor contributing to the differential cell response in in vitro models [38]. Melanin and the melanogenesis process itself exhibit a dual, opposing role in the biology of melanocytes and melanoma, referred to as the “Yin and Yang” effect. Under physiological conditions, melanogenesis primarily serves a protective function, whereas under pathological circumstances, it can promote melanoma cell survival, adaptation, and progression [42]. In healthy melanocytes, the melanogenesis process is tightly regulated and occurs within specialized cellular organelles (melanosomes). Melanin, especially eumelanin, plays an important photoprotective role by absorbing UV radiation and thus in limiting associated cellular damage. Additionally, eumelanin exhibits antioxidant properties, allowing it to neutralize reactive oxygen species (ROS) and limit their potentially harmful effects on DNA and other cellular components [37,38,39,40,41,42,43,44,45,46,47,48].

This phenomenon results from the presence of both oxidized and reduced melanin subunits [42]. In pathological conditions, however, melanin and melanogenesis may support cancer cell survival, modulate the tumor microenvironment and contribute to treatment resistance. Its dysregulation leads to the release of intermediate products of melanin synthesis outside melanosomes, including free radicals and highly reactive quinone compounds. These compounds can exhibit mutagenic, cytotoxic and genotoxic effects, and can also affect the signaling pathways and function of both melanoma cells and the cells within their microenvironment. Moreover, melanin’s ability to bind cytotoxic substances may also affect some anticancer drugs, potentially limiting their efficacy [44]. Additionally the process of melanogenesis, among others, by contributing to increased oxygen consumption, may influence tumor cell metabolism through the activation of HIF-1α. L-DOPA, an intermediate product of melanogenesis, can also enhance the activity of the glycolytic pathway in melanoma cells. HIF-1α is a key regulator of cancer cell adaptation to hypoxia and plays a significant role in the development of their resistance to treatment. Overexpression of HIF-1α directly promotes angiogenesis, migration, and the invasion of cancer cells, and also enhances anaerobic glucose metabolism. These changes may facilitate melanoma cell adaptation to metabolic stress and hypoxia, thus creating conditions favorable to cancer progression [45]. Furthermore, increased melanogenesis and the release of its metabolites may contribute to the creation of an immunosuppressive tumor microenvironment, for example, by inhibiting lymphocyte proliferation and activity and modulating cytokine production [46]. Therefore, the inhibition of melanogenesis is being considered as a potential adjunctive strategy in the treatment of melanotic melanomas, potentially increasing the sensitivity of tumor cells to chemotherapy, radiotherapy, and immunotherapy [42,47].

It is worth noting that other authors have also reported the antitumor properties of propolis, including anti-melanoma activity. Bidabad et al. also investigated the anticancer activity of liposome-encapsulated propolis nanoparticles from Iran using A375 cells. They demonstrated that liposome-encapsulated propolis possessed concentration- and time-dependent cytotoxic activity against A375 cells. The IC_50_ determined after 24 and 48 h using the MTT assay was 44 and 20 μg/mL, respectively. Moreover, it was shown that nanopropolis also inhibited the migration of A375 cells. At the same time, propolis was nontoxic to human dermal fibroblasts (HDFs) [48]. Oliveira et al. also observed decreased viability and increased ROS production in A375 (IC_50_ 16.98 ± 0.93 μg/mL) and WM9 melanoma cells (IC_50_ 25.03 ± 1.34 μg/mL) after treatment with Portuguese propolis [11]. Hochheim et al., on the other hand, determined the IC_50_ of dichloromethane propolis extract for mouse melanoma cells (B16-F10) and mouse normal fibroblasts (BALB/3T3), which were 36.15 μg/mL and 61.78 μg/mL, respectively, giving high SI > 1. It was also noted that the extract at a concentration of 15 μg/mL inhibited the migration of B16-F10 cells, without a negative effect on the migration of normal fibroblasts [49]. González-Masís et al. also confirmed the antimelanoma activity of propolis in the form of nanoparticles at a concentration of 5 µg/mL against the B16-F10 cell line [50]. Other authors also noted the cytotoxic activity of propolis against other tumors. Sezen et al. demonstrated that *Cistus laurifolius* originated propolis had an antitumor effect on pancreatic tumor cells (MIA PaCa-2), which was observed in the 2D and 3D model [51]. Durmaz et al. performed extensive screening and determined IC_50_ and SI values after 24-h incubation of propolis from Turkey with colorectal cancer cells (SW-620 cell line: IC_50_ = 345 μg/mL, SI = 0.85), prostate cancer cells (DU-145 cell line: IC_50_ = 189.4 μg/mL, SI = 1.54; PC-3 cell line: IC_50_ = 101.2 μg/mL, SI = 2.89), breast cancer (MCF-7 cell line: IC_50_ = 240.8 μg/mL, SI = 1.22) and normal fibroblasts (WI-38 cell line: IC_50_ = 292.6 μg/mL) [52]. Although cell culture models are valuable tools for initial screening, they do not fully reflect the complexity of living systems. Therefore, in vivo studies will be necessary to confirm whether the cytotoxic effects of propolis extracts observed in melanoma cells can be reproduced under physiological conditions, as well as to assess their safety [53,54,55,56,57,58,59,60,61,62,63,64,65].

Propolis is a chemically complex natural product containing numerous phenolic compounds, flavonoids, and other bioactive molecules, the concentrations of which vary depending on geographic and botanical origin. Several studies suggested that the presence of compounds such as artepillin C, chrysin, and caffeic acid phenethyl ester (CAPE) in propolis extract is responsible for its anticancer activity [53,54]. Statistical analyses excluded correlation between general composition (TP, TF) and IC_50_ against all cell lines (Table 3). Observed results suggest that the activity of propolis samples depended on their specific composition. The chemopreventive and antitumor activities of propolis are widely attributed to its rich content of polyphenolic and flavonoid constituents. Unfortunately, most of the chemical compounds present in NE-TRI propolis with the highest cytotoxicity against cancer cells could not be identified. Nevertheless, these were selected as the most promising Georgian propolis samples contained characteristic groups of compounds typically associated with this natural product, particularly flavonoids and their derivatives, including chrysin, pinocembrin, pinocembrin-3-acetate, apigenin, kaempferol, pinobanksin-5-methyl ether, and luteolin-5-methyl ether. In addition, the extracts were rich in free phenolic acids, such as caffeic acid and *p*-coumaric acid, as well as their corresponding esters. Identified ester derivatives included caffeic acid 2-methyl-2-butenyl ester, caffeic acid 3-methyl-2-butenyl ester, *p*-coumaric acid cinnamyl ester, caffeic acid phenethyl ester (CAPE), and *p*-coumaric acid 3-methyl-2-butenyl ester. These compounds represent key constituents responsible for the chemical profile of Georgian propolis extracts. It is in agreement with the available literature, indicating that the presence of chrysin and CAPE (caffeic acid phenethyl ester) in propolis is responsible for its antitumor activity. The selected Georgian propolis samples with the highest selectivity index (GE-OR, GE-Q2, GE-VA1) contained both of these compounds in varying amounts. The phenolic compound CAPE has been identified as the main active component of propolis. The antitumor activity of CAPE was demonstrated using multiple cell lines, including oral cancer cells (OSF, GNM, TSCCa), human ovarian cancer (SK-OV-3), human lung cancer (NCI-H358), human hepatocellular carcinoma (HepG2), and glioma (C6). Its pro-apoptotic effects involved both intrinsic and extrinsic pathways. Application of CAPE increased the expression of pro-apoptotic proteins (Bax, Bak, p53, p21) and decreased the expression of anti-apoptotic proteins such as Bcl-2 and IAP, while promoting cytochrome c release and caspase activation. These effects appear to be largely independent of the tumor type or CAPE concentration [55,56,57,58,59,60,61]. Chrysin (5,7-dihydroxyflavone) is a natural flavonoid found in honey and propolis. The mechanisms of its antitumor activity have not been fully elucidated. Chrysin promoted apoptosis in various tumor cell lines by inhibiting the PI3K/Akt and NF-κB pathways, reducing anti-apoptotic proteins (IAP, c-FLIP), and activating caspases, particularly caspase-3 and caspase-8. It also induced the release of mitochondrial cytochrome c, indicating involvement in the intrinsic apoptotic pathway. Furthermore, chrysin enhanced TNF-α-induced apoptosis in many tumor cells [60,62,63,64,65].

### 2.4. Qualitative Assessment of Apoptosis and Necrosis in Propolis-Treated Cells

Based on a cytotoxicity test, propolis samples with the highest S_I_ values (GE-OR, GE-Q2, and GE-VA1) were selected for the assessment of the type of cell death pathway (apoptosis, necrosis). Apoptosis, also known as programmed cell death, is a process of controlled, sequential events leading to cell elimination. This process is characterized by specific morphological features. Apoptosis participates in maintaining body homeostasis and occurs under normal physiological conditions during aging and development [66]. Necrosis is a passive, uncontrolled, and energy-independent process leading to cell death. During necrosis, cell membrane integrity is lost, and cytoplasmic contents are released into the surrounding environment, leading to inflammation [66].

**Figure 3:**
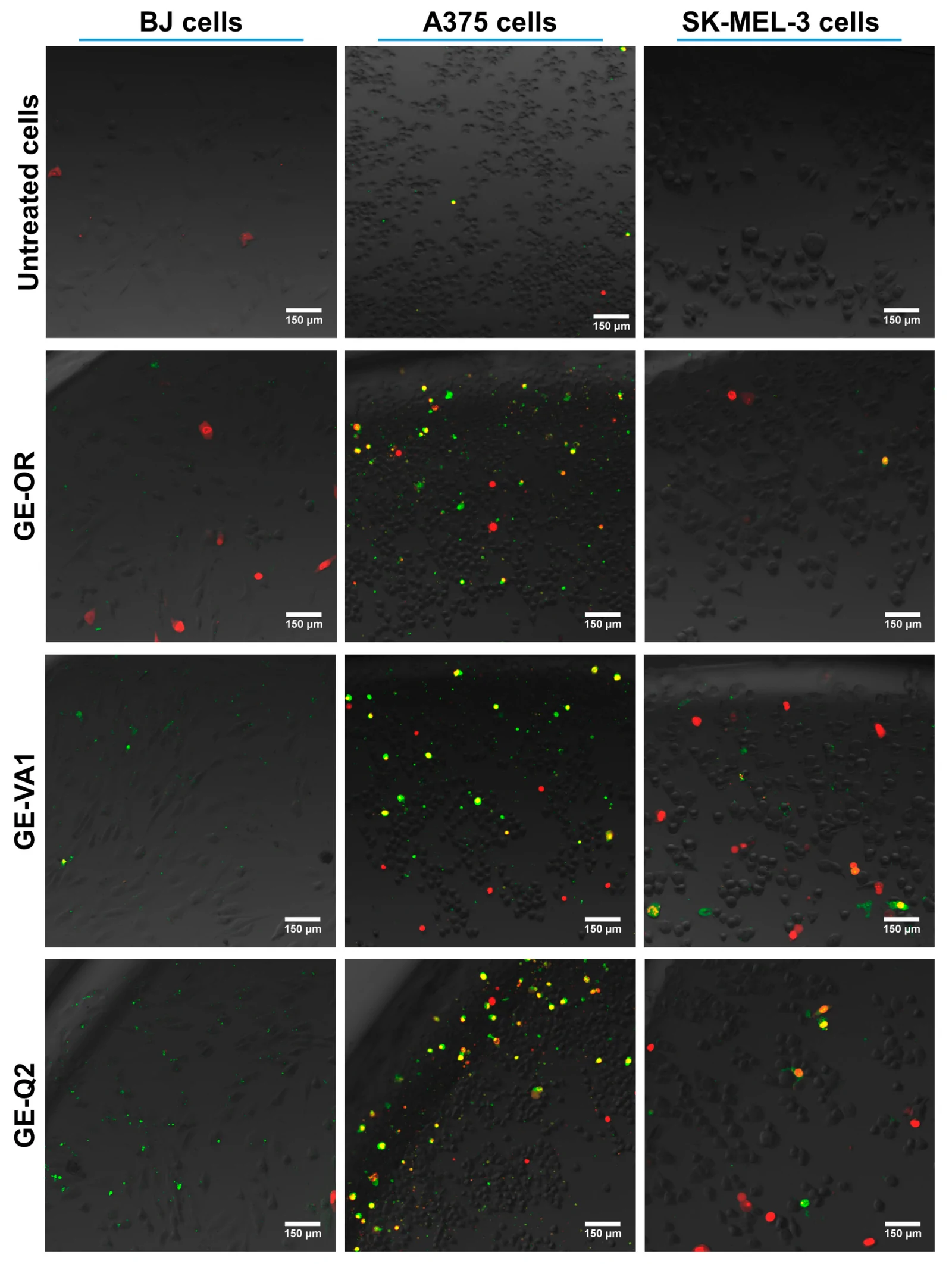
CLSM images of cells after 24-h treatment with selected propolis samples at their IC_50_ concentrations. The cells were fluorescently stained with the Apoptosis/Necrosis Assay Kit (green fluorescence—early apoptotic cells, yellow fluorescence—late apoptotic cells, red fluorescence—necrotic cells).

The study results showed that the highest amounts of early and late apoptotic cells (green and yellow fluorescence) were observed after exposure of the A375 melanoma cells to extracts of the selected propolis samples (Figure 3). Confocal microscopy images also demonstrated a few necrotic cells (red fluorescence). All three propolis extracts resulted in comparable amounts of dead cells. After incubation with the GE-VA1 and GE-Q2 propolis extracts, a few dead cells, mostly necrotic (red signal) and single apoptotic (green and yellow signal), were observed in the SK-MEL-3 cell line. Images of the BJ cell line after treatment with the GE-OR propolis extract revealed a small number of necrotic cells (red signal). In contrast, exposure to the GE-VA1 and GE-Q2 extracts resulted in the presence of a few apoptotic cells. It is consistent with studies by Bidabad et al., who speculated that propolis from Iran exerted its toxicity on tumor cells by inducing early and late apoptosis. The authors observed an increase in the number of apoptotic A375 cells in flow cytometry analysis after exposure to the IC_50_ of liposome-encapsulated nanopropolis [48].

On the other hand, Orsolic and Basic observed different responses of cell lines exposed to propolis extracts. They investigated the effect of water-soluble propolis derivatives from Croatia and Brazil on the induction of apoptosis in Chinese hamster lung fibroblasts (V79 cell line), human epithelial carcinoma cell line (HeLa cell line), and mammary gland carcinoma cells (MCA cell line). After 15 h of incubation with 50 μg/mL of Croatian and Brazilian propolis, the percentage of apoptotic MCA cells increased to 24% and 26%, respectively, compared to the control (20%). In the case of HeLa cells, an increase in the percentage of apoptotic cells to 10% and 9.5% compared to the control (2%) was observed after exposure to the extracts of Croatian and Brazilian propolis, respectively. In contrast, in the V79 cells, a decrease in the percentage of apoptotic cells compared to the control was detected after treatment with the propolis extracts [67].

Importantly, the type of cell death pathways (apoptosis or necrosis) in some cases depends not only on the type of stimulus, but also on its intensity. The same harmful factor at low concentrations can lead to cell apoptosis, whereas at higher doses, it can induce necrosis [66]. A crucial element of cell death is the presence of inflammation [68]. During apoptosis, cells are fragmented into small membrane-bound vesicles, which are phagocytosed by other cells. Importantly, apoptosis is not accompanied by an inflammatory reaction and does not lead to damage to surrounding cells. Necrosis is a proinflammatory type of cell death that usually spreads rapidly to neighboring cells, leading to their damage [68,69,70]. Apoptosis is generally considered the preferred form of cell death due to the lack of an inflammatory response. However, proinflammatory forms of cell death may also have important functions. Necrosis can lead to the widespread cell death of entire populations of damaged or cancerous cells by recruiting immune cell [70].

The performed analyses provided preliminary information on potential cell death pathways; however, they did not allow for a comprehensive characterization of the molecular pathways involved. More detailed studies are planned, including broader quantitative analyses of the mechanisms of cell death, oxidative stress responses, and cell cycle changes.

### 2.5. Assessment of Caspase-6 Activity in Propolis-Treated Cells

**Figure 4:**
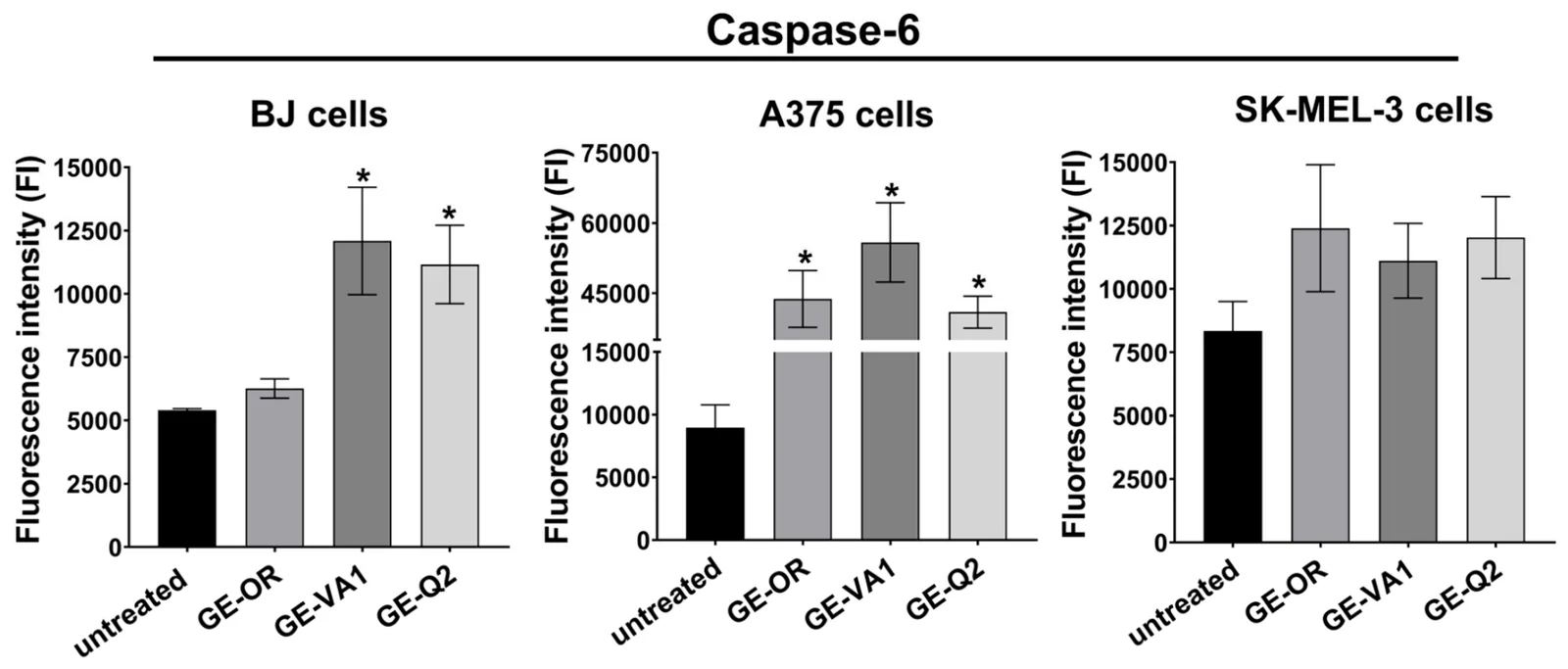
Assessment of caspase-6 levels in cells after 48-h treatment with selected propolis samples at their IC_50_ concentrations (statistical analysis: *p* < 0.05, one-way ANOVA with Dunnett’s test; * indicates statistically significant differences compared with the corresponding untreated control cells).

During apoptosis, caspase-6 is activated in the cell nucleus. This caspase is responsible for chromosomal DNA breakage and nuclear atrophy by cleaving various transcription factors and structural proteins, among others. Caspase-6 is activated via caspase-7 and caspase-3, and conversely, activates caspase-7 and caspase-3. Therefore, the modulation of apoptosis via caspase-6, which functions as both an initiator and an executioner within the apoptotic pathway, is of particular significance [71]. The highest caspase-6 activity was observed in A375 melanoma cells after exposure to extracts from all selected propolis samples (Figure 4).

Caspase-6 activity in propolis-treated A375 melanoma cells was significantly higher compared to the untreated control cells and ranged from 40,922 ± 3413 FI (GE-Q2) to 55,860 ± 8500 FI (GE-VA1). This may indicate that cell death occurred primarily via apopotosis, which was consistent with the results of fluorescent cell staining. In the case of normal BJ cells and SK-MEL-3 melanoma cells, fluorescence intensity measurements showed comparable levels of caspase-6 activity after exposure to GE-VA1 and GE-Q2 propolis extracts, with values of 12,091 ± 2123 FI (BJ), 11,112 ± 1477 FI (SK-MEL-3), and 11,162 ± 1550 FI (BJ), 12,027 ± 1621 FI (SK-MEL-3), respectively. In contrast, the lowest caspase-6 activity was observed after the exposure of BJ cells to GE-OR propolis (6263 ± 381 FI) and was comparable to the values for control cells, which may specify cell death by a pathway other than apoptosis. It is also worth noting that the level of caspase-6 in untreated (control) normal cells (5402 ± 67 FI) was lower than in untreated melanoma cells (approx. 8500 FI). Oliveira et al. showed that Portuguese propolis extract and its fractions significantly increased the level of caspase-9 in A375 and WM9 melanoma cell lines, as well as decreased the level of antiapoptotic protein Bcl-XL in A375 cells [11]. Other authors observed an increase in the expression of the proapoptotic genes (caspase-3, BAX) and a decrease in the expression of the antiapoptotic Bcl-2 in the A375 cell line exposed to propolis nanoparticles from Iran [48]. Durmaz et al. examined, in several propolis-treated tumor cell lines and normal fibroblasts (WI-38), the levels of apoptosis markers: caspase-3, -8, and -9, as well as ligands associated with apoptosis induction: tumor necrosis factor (TRAIL) and apoptosis protease-activating factor 1 (APAF-1). A statistically significant 3.62-fold increase in caspase-3 levels and 4.38-fold increase in TRAIL levels were demonstrated in the colorectal cancer cells (SW-620 cell line) compared to normal WI-38 cells. In the prostate cancer cells (PC-3 cell line), statistically significant 1.4-fold increase in caspase-8 level was observed compared to normal WI-38 cells [52]. Statistical analyses excluded correlation between general composition (TP, TF) and the activity of caspase-6 (Table 5). This result suggests that general level of activity was a result of the interaction between different components rather than simply the amount of polyphenols and flavonoids. Further investigation is required to explain this phenomenon.

## 3. Materials and Methods

### 3.1. Propolis and Preparation of the Extracts

Propolis samples were collected from the endemic areas of Nepal, Greece, Ukraine, Kazakhstan, and Georgia. The exact regions and year of collecting are listed in Table 1. Most of the samples were produced by *Apis mellifera* L., while the sample from Nepal was obtained from *Trigona* spp.

Extraction process was performed as described previously [29]. In brief, the solution of 96% ethanol in water (70:10 *v*/*v*) was used to extract the grounded research material. Every extract was obtained from a dried portion of propolis material. Extraction was performed in an ultrasonic bath (Sonorex, Bandelin, Berlin, Germany) at 20 °C (initial temperature) for 15 min and 756 W (90% of ultrasonic bath power). Process was repeated three times (full time of extraction was 45 min). Before each cycle, extracts were mixed and the temperature of ultrasound bath was controlled—for our purposes, this was maintained at a temperature under 50 °C due to avoiding potential polyphenol destruction. After the third cycle, the temperature was 45 °C. Obtained extracts were stored at room temperature for 12 h for stabilization purposes (precipitation of potentially dissolved wax from extracts). Next, extracts were filtered through Whatman No. 10 paper (Cytiva, Marlborough, MA, USA) and ethanol was evaporated from the extracts under lower pressure. Then, frozen extracts were lyophilized in Christ Alpha 2–4 LD Plus lyophilizer (Christ, Osterode am Harz, Germany).

### 3.2. UHPLC-UV-ESI-qTOF-MS/MS Profile of Propolis Extracts

LiChrosolv^®^ hypergrade eluents for UHPLC-MS/MS and UHPLC-DAD analysis (acetonitrile, water, and methanol) were purchased from Merck (Darmstadt, Germany). Standards for acacetin, apigenin, chrysin, kaempferol, kaempferide, isorhamnetin, isosakuranetin, luteolin, genkwanin, pinocembrin, pinocembrin chalcone, pinocembrin dihydrochalcone, pinobanksin-, pinostrobin, quercetin, rhamnetin, sakuranetin, and tectochrysin were purchased from Extrasynthese (Genay, France), while caffeic acid, caffeic acid phenethyl ester (CAPE), and ferulic acid were obtained from Sigma-Aldrich (Saint Louis, USA). UHPLC-UV-ESI-qTOF-MS/MS analyses were performed as described previously [29] using a Thermo Scientific UltiMate 3000 system (Thermo Scientific™ Dionex™, Sunnyvale, CA, USA), coupled with an autosampler and DAD detector recording spectral data in the 200–600 nm range and monitoring at 280, 320 and 360 nm. UHPLC-MS/MS was carried out using a Compact ESI-qTOF-MS/MS detector (Bruker Daltonics, Bremen, Germany).

Identification of components was performed based on several parameters such as the retention times of chromatographic peaks, UV spectra, calculated formulas of deprotonated molecular ions as well as MS/MS fragmentation spectra of deprotonated molecular ions. Due to information collected from the literature, four levels of identification confidence were obtained: A (comparison of UV and MS/MS spectra with standards; the highest level of confidence), B (comparison of MS/MS and/or UV spectrum with literature; good level of confidence), C (component was identified according to deprotonated molecular ion formula and prediction from MS/MS spectra detected in the propolis genus literature, but there were not sufficient MS and UV data; average/weak level of confidence), and D (component was identified according to deprotonated molecular ion and prediction from MS spectra, but there were not sufficient MS/MS and UV data and substances were not reported in the propolis genus literature; the weakest level of confidence). Components that had a deprotonated molecular ion formula calculation error higher than ∆ 5 ppm were not included in the research.

Half-quantitative analysis was based on the relative abundance of components in the UV chromatogram (280 nm) and MS chromatogram. Relative abundance of most components was obtained due to deprotonated molecular ion intensity in a single chromatographic peak (IDMI). Due to received intensity, eight levels of relative abundance were created: tr (IDMI < 5 × 10^4^); + (5 × 10^4^ < IDMI > 1.5 × 10^5^); ++ (1.5 × 10^5^ < IDMI > 3 × 10^5^); +++ (3 × 10^5^ < IDMI > 4.5 × 10^5^); ++++ (4.5 × 10^5^ < IDMI > 6.0 × 10^5^); +++++ (6.0 × 10^5^ < IDMI > 7.5 × 10^5^); ++++++ (7.5 × 10^5^ < IDMI > 1.0 × 10^6^); +++++++ (1.0 × 10^6^ < IDMI). Only pinostrobin and tectochrysin relative amounts were based on UV peak intensity due to their weak ionization in negative mode. Due to received intensity, seven levels of relative abundance for UV chromatograms were created: tr (height < 1 mAU); + (1< height >5 mAU); ++ (5< height >10 mAU); +++ (10< height >15 mAU); ++++ (15< height >20 mAU); +++++ (20< height >25 mAU); ++++++ (25< height mAU).

### 3.3. Total Flavonoid (TF), Total Phenolic (TP) Content and Antioxidant Potential (DPPH and FRAP Assays)

Lyophilized extracts were dissolved in methanol to obtain stock solutions of 1 mg/mL. These stock solutions were then diluted from 2 to 32 times, and preliminary tests were performed to determine the appropriate dilution for each assay [31].

#### 3.3.1. Total Flavonoid (TF), and Total Phenolic (TP) Content

The total phenolic (TP) content of the propolis extracts was determined spectrophotometrically using the Folin–Ciocalteu method, following previously described procedures [72,73]. Prior to analysis, the extracts were diluted 20- to 200-fold. An aliquot of 50 μL of each ethanolic extract was mixed with 20 μL of Folin–Ciocalteu reagent. After 5 min, 200 μL of sodium carbonate (Na_2_CO_3_) solution (100 g/L) was added, and the reaction mixtures were incubated for 90 min at room temperature in the dark. Subsequently, the absorbance was measured at 725 nm using disposable polystyrene 96-well plates and a microplate spectrophotometer. A reagent blank was prepared following the same procedure, with the sample replaced by the corresponding pure solvent. The TP content was quantified using a calibration curve generated from freshly prepared gallic acid standard solutions at concentrations ranging from 10 to 200 μg/mL. The results were expressed as milligrams of gallic acid equivalents per gram of propolis (mg GAE/g). All measurements were performed in triplicate. The total flavonoid (TF) content of the propolis extracts was determined spectrophotometrically using a modified pharmacopoeial method based on the formation of aluminum-flavonoid complexes with aluminum chloride (AlCl_3_). Briefly, 50 μL of each prepared extract was mixed with 50 μL of a 2% (*w*/*v*) ethanolic AlCl_3_ solution. The reaction mixtures were incubated for 60 min at room temperature in the dark. Subsequently, the absorbance was measured at 420 nm using a microplate reader. The TFC was quantified using a calibration curve prepared with quercetin standard solutions over the concentration range of 20–400 μg/mL. The results were expressed as milligrams of quercetin equivalents per gram of propolis (mg QE/g). All measurements were performed in triplicate.

#### 3.3.2. Antiradical Activity (DPPH Assay)

The antiradical activity of the propolis extracts was determined using the DPPH (2,2-diphenyl-1-picrylhydrazyl) radical-scavenging assay, with gallic acid used as the reference standard, according to a previously modified method [74]. Prior to analysis, the propolis extracts were diluted 20- to 200-fold. Subsequently, 20 µL of each diluted extract was mixed with 200 µL of 0.315 mM DPPH solution prepared in methanol. The mixtures were incubated for 30 min at room temperature in the dark. Absorbance was measured at 517 nm using disposable optical polystyrene 96-well microplates and a microplate spectrophotometer, as described previously. All measurements were performed in triplicate. The antiradical activity was quantified using a gallic acid calibration curve constructed over the concentration range of 2.0–100 µg/mL and expressed as milligrams of gallic acid equivalents per gram of propolis (mg GAE/g).

#### 3.3.3. Total Antioxidant Activity (FRAP Assay)

The ferric reducing antioxidant power (FRAP) assay was performed according to previously described methods [75,76]. Briefly, the FRAP reagent was prepared by combining 10 mmol/L TPTZ (2,4,6-tri(2-pyridyl)-s-triazine) with 20 mmol/L ferric chloride in acetate buffer (pH 3.6). Quantification was performed using a calibration curve prepared with ferrous sulfate as the external standard over the concentration range of 0.02–1.5 μmol/mL. Prior to analysis, the propolis extracts were diluted 20- to 200-fold. An aliquot of 20 μL of each diluted extract was then mixed with 200 μL of the FRAP reagent. Absorbance was measured at 593 nm using disposable optical polystyrene 96-well plates (FL Medical, Torreglia, Italy) and a Multiskan™ GO Microplate Spectrophotometer (Thermo Fisher Scientific, Waltham, MA, USA). The antioxidant capacity was calculated from the ferrous sulfate calibration curve and expressed as mmol Fe^2+^ per gram of propolis. All measurements were performed in triplicate.

### 3.4. Anticancer Activity of Propolis Extracts Against Melanoma Cells

#### 3.4.1. Cell Culture

Evaluation of the cytotoxicity of propolis extracts was performed using human normal skin fibroblasts (BJ cell line, CRL-2522, ATCC-LGC Standards, Teddington, UK) and malignant melanoma cells (A375 cell line, CRL-1619 and SK-MEL-3 cell line, HTB-69, ATCC-LGC Standards, Teddington, UK). BJ cells were cultured in Eagle’s minimum Essential medium (ATCC-LGC Standards, Teddington, UK) supplemented with 10% fetal bovine serum (FBS, Pan-Biotech GmbH, Aidenbach, Bavaria, Germany) and 1% penicillin–streptomycin solution (Sigma-Aldrich Chemicals, Warsaw, Poland). A375 cells were cultured in Dulbecco’s modified Eagle’s medium (ATCC-LGC Standards, Teddington, UK) supplemented with 10% FBS and 1% penicillin–streptomycin solution. SK-MEL-3 cells were cultured in McCoy’s 5a Medium Modified (ATCC-LGC Standards, Teddington, UK) supplemented with 15% FBS and 1% penicillin–streptomycin solution. BJ, A375, and SK-MEL-3 cells were maintained at 37 °C in a 5% CO_2_ air atmosphere.

#### 3.4.2. Cytotoxicity Assay

The cytotoxicity of the propolis extracts against normal skin cells and malignant melanoma cells was determined by the MTT assay (Sigma-Aldrich Chemicals, Warsaw, Poland). Serial twofold dilutions of the propolis extracts (ranging from 0.98 to 1000 μg/mL) in the appropriate cell medium were analysed. First, 100 μL of the BJ, A375, and SK-MEL-3 cells at a concentration of 2 × 10^5^ cells/mL were seeded into 96-well plates. After 24 h of culture, the medium was replaced with 100 μL of different concentrations of the tested propolis extracts. After 24 h of exposure to the propolis extracts, cell viability was evaluated using the MTT assay as described previously [77]. The results of the MTT assay were expressed as the percentage of the absorbance value obtained with the negative cytotoxicity control (cells cultured in growth medium without propolis extracts), which was considered to represent 100% viability. The concentration of propolis extract that reduced cell viability by 50% (IC_50_ value) was determined using GraphPad Prism 8 software (version 8.0.0; GraphPad Software Inc., San Diego, CA, USA). Additionally, the selectivity/safety index (SI) was calculated using the following formula: SI = IC_50_ of propolis in normal cells/IC_50_ of the same propolis in the cancer cell line.

#### 3.4.3. Qualitative Assessment of Apoptosis and Necrosis

BJ, A375, and SK-MEL-3 cells were seeded into 96-well plates at a density of 2 × 10^5^ cells/mL (100 μL per well). After 24 h of culture, the medium was replaced with 100 μL of the selected propolis extracts at their respective IC_50_ concentrations. Cells cultured in medium without propolis served as control/untreated cells. Following 24 h of exposure, the cells were fixed with 3.7% (*v*/*v*) paraformaldehyde (Sigma-Aldrich Chemicals, Warsaw, Poland) and subjected to fluorescent staining using the Apoptosis/Necrosis Assay Kit (Abcam, Cambridge, UK) containing Apopxin Green (green fluorescence, stains apoptotic cells) and 7-AAD (red fluorescence, stains late apoptotic and necrotic cells). The stained cells were observed using a confocal laser scanning microscope (CLSM, Olympus Fluoview FV1000, Olympus Corporation, Tokyo, Japan).

#### 3.4.4. Assessment of Caspase-6 Levels

BJ, A375, and SK-MEL-3 cells were seeded into 96-well plates at a density of 2 × 10^5^ cells/mL (100 μL per well). After 24 h of culture, the medium was replaced with 100 μL of selected propolis extracts at their respective IC_50_ concentrations. Cells cultured in medium without propolis served as control/untreated cells. Following 48 h of exposure, caspase-6 levels were measured using a Fluorometric Caspase-6 Assay Kit (FAM-FLICA Caspase Assays, ImmunoChemistry Technologies, LLC, California, USA) according to the manufacturer’s protocol. Caspase-6 activity was presented as fluorescence intensity (FI) values.

### 3.5. Statistical Analysis

Data from the conducted tests were presented as the mean and standard deviation (SD) from at least three independent repetitions of the experiment. In the cytotoxicity test, statistically significant differences between control cells and cells exposed to individual concentrations of propolis were considered at *p*  <  0.05 using one-way ANOVA analysis, with Dunnett’s post hoc test. In the remaining tests, to detect statistically significant differences between the samples, one-way ANOVA, followed by the Tukey test was applied, with statistically significant results considered at *p*  <  0.05 (GraphPad Prism 8.0.0 software, GraphPad Software Inc., CA, USA). Connection between biological activities (DPPH, FRAP, IC_50_ against cell lines and assessment of caspase-6 levels) and general composition (TP and TF) was analyzed using Statistica 14.0.0.15 software (Tibco Software Inc., Palo Alto, USA). Analyses based on correlation matrices with the calculation of Pearson correlation and R parameters were applied. Input data used to perform the correlation test are presented in Supplementary Materials Tables S3 and S4.

## 4. Conclusions

The main aim of this study was to perform a preliminary screening of a large collection of endemic propolis samples to identify extracts with potentially selective cytotoxic activity against melanoma cells. The assays used in this research provided an effective strategy for narrowing down the number of candidates for further investigation. Among the tested samples, propolis samples from endemic regions of Georgia (Orgora—GE-OR sample, Vazisuban—GE-VA1 sample, and Qvakhvreli—GE-Q2 sample) showed the strongest cytotoxic activity against the A375 and SK-MEL-3 melanoma cell lines while maintaining low toxicity towards normal human skin fibroblasts (BJ cell line). These effects were accompanied by a high polyphenol content and strong antioxidant capacity. Confocal laser scanning microscope analysis revealed cell line-dependent responses, with apoptosis predominating in A375 cells and necrosis in SK-MEL-3 cells, supported by high caspase-6 activity in A375 cells. Given its reported anti-melanoma activity and regenerative potential, the topical application of Georgian propolis to the surgical wound following melanoma excision may represent a beneficial therapeutic approach.

## Figures and Tables

**Table 1:** Origin of tested propolis samples.

Abbreviations	Country	Region and Year of Collecting	Geographical Coordinates of the Region
NE-TRI	Nepal	Korak village, 2016	27.68391 °N, 84.71722 °E
GR-PA	Greece	Parga, 2020	39.28572 °N, 20.40044 °E
KA-BO	Kazakhstan	Bozovoe, 2021	46.21828 °N, 58.82584 °E
UK-TA	Ukraine	Tarnopol, 2020	49.55404 °N, 25.59067 °E
GE-OR	Georgia	Orgora, 2021	41.57890 °N, 43.19078 °E
GE-OT	Georgia	Ota, 2021	41.61966 °N, 43.30174 °E
GE-Q1	Georgia	Qvakhvreli, 2021	41.95898 °N, 44.21711 °E
GE-Q2	Georgia	Qvakhvreli, 2022	41.95898 °N, 44.21711 °E
GE-VA1	Georgia	Vazisubani, 2021	41.82331 °N, 45.69528 °E
GE-VA2	Georgia	Vazisubani, 2022	41.82331 °N, 45.69528 °E

**Table 2:** Colorimetric evaluation of total phenolic (TP) and flavonoid (TF) content, antioxidant and antiradical activity of the propolis extracts.

Sample Abbreviation	DPPH [mgGAE/g Extract]	FRAP [mmolFe^2+^/g Extract]	TF [mgQUE/g Extract]	TP [mgGAE/g Extract]
NE-TRI	41.06 ± 0.79	6.82 ± 0.13	34.79 ± 1.71	116.21 ± 3.03
GR-PA	67.27 ± 1.53 ^a^	12.72 ± 0.02 ^a^	219.69 ± 1.60 ^a^	218.72 ± 0.92 ^a^
KA-BO	44.59 ± 0.20 ^b^	11.80 ± 0.21 ^a^	119.34 ± 1.85 ^a,b^	184.95 ± 3.46 ^a,b^
UK-TA	64.45 ± 0.01 ^a,c^	13.92 ± 0.38 ^a,b,c^	191.14 ± 1.49 ^a,b,c^	168.35 ± 1.17 ^a,b,c^
GE-OR [29]	117.71 ± 1.04 ^a,b,c,d^	13.76 ± 0.15 ^a,c^	96.07 ± 4.73 ^a,b,c,d^	193.06 ± 2.55 ^a,b,c,d^
GE-OT [29]	71.26 ± 0.60 ^a,c,d,e^	13.50 ± 0.31 ^a,c^	122.07 ± 1.56 ^a,b,d,e^	242.71 ± 3.12 ^a,b,c,d,e^
GE-Q1 [29]	116.38 ± 1.50 ^a,b,c,d,f^	16.83 ± 1.02 ^a,b,c,d,e,f^	81.50 ± 1.44 ^a,b,c,d,e,f^	218.70 ± 4.94 ^a,c,d,e,f^
GE-Q2	111.06 ± 4.38 ^a,b,c,d,e,f,g^	19.09 ± 0.04 ^a,b,c,d,e,f,g^	136.15 ± 1.68 ^a,b,c,d,e,f,g^	286.77 ± 1.74 ^a,b,c,d,e,f,g^
GE-VA1	88.71 ± 0.21 ^a,b,c,d,e,f,g,h^	13.75 ± 0.22 ^a,c,g,h^	106.80 ± 0.22 ^a,b,c,d,e,f,g,h^	227.55 ± 1.63 ^a,b,c,d,e,f,g,h^
GE-VA2	20.94 ± 0.07 ^a,b,c,d,e,f,g,h,i^	9.42 ± 0.12 ^a,b,c,d,e,f,g,h,i^	56.08 ± 0.46 ^a,b,c,d,e,f,g,h,i^	102.18 ± 1.31 ^a,b,c,d,e,f,g,h,i^

Units acronyms: mgGAE/g extract—mg of gallic acid equivalents per gram of dry extract; mmolFe^2+^/g extract—mmol of Fe^2+^ equivalents per gram of dry extract; mgQUE/g extract—mg of quercetin equivalents per gram of dry extracts. Propolis extract acronyms: NE-TRI—Nepal, Trigona; GR-PA—Greece, Parga; KA-BO—Kazakhstan, Bozovoe; UK-TA—Ukraine, Tarnopol; GE-OR—Georgia, Orgora; GE-OT—Georgia, Ota; GE-Q1—Georgia, Qvakhvreli sample 1; GE-Q2—Georgia, Qvakhvreli sample 2; GE-VA1—Georgia, Vasisubani sample 1; GE-VA2—Georgia, Vasisubani sample 2. Test acronyms: DPPH—2,2-diphenyl-1-picrylhydrazyl radical scavenging activity; FRAP—ferric reducing antioxidant power assay. ^a^ statistically significant results compared to NE-TRI, ^b^ statistically significant results compared to GR-PA, ^c^ statistically significant results compared to KA-BO, ^d^ statistically significant results compared to UK-TA, ^e^ statistically significant results compared to GE-OR, ^f^ statistically significant results compared to GE-OT, ^g^ statistically significant results compared to GE-Q1, ^h^ statistically significant results compared to GE-Q2, ^i^ statistically significant results compared to GE-VA1; *p*-value < 0.05, one-way ANOVA followed by the Tukey post hoc test.

**Table 3:** Correlation matrix of general composition (TP, TF) and biological activities (DPPH, FRAP, half maximal inhibitory concentration (IC_50_)).

Variable	Correlations of Composition (TP, TF) and Biological Activities (DPPH, FRAP, IC_50_). Marked Correlations Are Significant at *p* < 0.05000 N = 10 (Casewise Deletion of Missing Data)						
	BJ	A375	SK-MEL-3	DPPH	FRAP	TF	TP
BJ	R^2^ = 1.0000	R^2^ = 0.0424	R^2^ = 0.5463	R^2^ = 0.0744	R^2^ = 0.3909	R^2^ = 0.3077	R^2^ = 0.4717
	*p* = ---	*p* = 0.908	*p* = 0.102	*p* = 0.838	*p* = 0.264	*p* = 0.387	*p* = 0.169
A375	R^2^ = 0.0424	R^2^ = 1.0000	R^2^ = 0.5650	R^2^ = −0.1615	R^2^ = 0.0374	R^2^ = 0.6273	R^2^ = −0.1397
	*p* = 0.908	*p* = ---	*p* = 0.089	*p* = 0.656	*p* = 0.918	*p* = 0.052	*p* = 0.700
SK-MEL-3	R^2^ = 0.5463	R^2^ = 0.5650	R^2^ = 1.0000	R^2^ = 0.0356	R^2^ = 0.2710	R^2^ = 0.3594	R^2^ = −0.0163
	*p* = 0.102	*p* = 0.089	*p* = ---	*p* = 0.922	*p* = 0.449	*p* = 0.308	*p* = 0.964
DPPH	R^2^ = 0.0744	R^2^ = −0.1615	R^2^ = 0.0356	R^2^ = 1.0000	**R^2^ = 0.8184**	R^2^ = 0.1520	**R^2^ = 0.7373**
	*p* = 0.838	*p* = 0.656	*p* = 0.922	*p* = ---	***p* = 0.004**	*p* = 0.675	***p* = 0.015**
FRAP	R^2^ = 0.3909	R^2^ = 0.0374	R^2^ = 0.2710	**R^2^ = 0.8184**	R^2^ = 1.0000	R^2^ = 0.4142	**R^2^ = 0.8589**
	*p* = 0.264	*p* = 0.918	*p* = 0.449	***p* = 0.004**	*p* = ---	*p* = 0.234	***p* = 0.001**
TF	R^2^ = 0.3077	R^2^ = 0.6273	R^2^ = 0.3594	R^2^ = 0.1520	R^2^ = 0.4142	R^2^ = 1.0000	R^2^ = 0.4699
	*p* = 0.387	*p* = 0.052	*p* = 0.308	*p* = 0.675	*p* = 0.234	*p* = ---	*p* = 0.171
TP	R^2^ = 0.4717	R^2^ = −0.1397	R^2^ = −0.0163	**R^2^ = 0.7373**	**R^2^ = 0.8589**	R^2^ = 0.4699	R^2^ = 1.0000
	*p* = 0.169	*p* = 0.700	*p* = 0.964	***p* = 0.015**	***p* = 0.001**	*p* = 0.171	*p* = ---

**Red color** indicates selected propolis samples for further biological studies. Units acronyms: P—Pearson’s correlation parameter; R^2^—regression parameter. Cell line and test acronyms: BJ—normal human fibroblast cell line; A375—primary malignant melanoma cell line; SK-MEL-3—highly invasive melanoma cell line originated from lymph; DPPH—2,2-diphenyl-1-picrylhydrazyl radical scavenging activity; FRAP—ferric reducing antioxidant power assay; TF—flavonoid content; TP—total polyphenol content.

**Table 4:** The IC_50_ values and the safety index (SI) of propolis derived from endemic areas.

Propolis	IC_50_ [μg/mL]			SI	
	BJ	A375	SK-MEL-3	A375	SK-MEL-3
NE-TRI	47.1 ± 2.8	81.3 ± 4.3	42.6 ± 1.1	0.58	1.11
GR-PA	157.5 ± 2.6 ^a^	203.6 ± 16.0 ^a^	138.6 ± 3.0 ^a^	0.77	1.14
KA-BO	166.6 ± 3.7 ^a^	201.4 ± 5.8 ^a^	131.5 ± 2.0 ^a^	0.83	1.27
UK-TA	112.0 ± 5.6 ^a,b,c^	262.7 ± 17.1 ^a,b,c^	173.9 ± 3.1 ^a,b,c^	0.43	0.64
GE-OT	132.2 ± 14.1 ^a^	188.1 ± 15.5 ^a,d^	145.3 ± 2.8 ^a,d^	0.70	0.91
**GE-OR**	174.8 ± 22.7 ^a,d,e,f^	146.6 ± 2.7 ^a,b,c,d^	130.1 ± 9.4 ^a,d,e,f^	**1.19**	**1.34**
GE-Q1	121.2 ± 5.5 ^a,b,c^	124.1 ± 8.6 ^a,b,c,d,e^	147.8 ± 2.4 ^a,d^	0.98	0.82
**GE-Q2**	162.5 ± 13.3 ^a,d,f^	114.3 ± 16.8 ^b,c,d,e,g^	101.2 ± 2.5 ^a,b,c,d,e,f,g^	**1.42**	**1.61**
**GE-VA1**	183.0 ± 4.6 ^a,d,e,f^	59.9 ± 3.7 ^b,c,d,e,f^	138.6 ± 9.9 ^a,d^	**3.06**	**1.32**
GE-VA2	172.4 ± 6.3 ^a,d,e^	154.8 ± 6.4 ^a,b,d,g^	175.6 ± 2.7 ^a,b,c,f,g^	1.11	0.98

**Red color** indicates selected propolis samples for further biological studies. Statistical analysis was performed in the appropriate cell line: *p* < 0.05, one-way ANOVA followed by Tukey’s post hoc test; ^a^ indicates statistically significant differences compared with NE-TRI; ^b^ indicates statistically significant differences compared with GR-PA; ^c^ indicates statistically significant differences compared with KA-BO; ^d^ indicates statistically significant differences compared with UK-TA; ^e^ indicates statistically significant differences compared with GE-OT; ^f^ indicates statistically significant differences compared with GE-Q1; ^g^ indicates statistically significant differences compared with GE-VAC1.

**Table 5:** Correlation matrix of general composition (TP, TF) and biological activity (caspase-6 level).

Variable	Correlations. Composition (TP, TF) and Biological Activity (Caspase 6 level). Marked Correlations Are Significant at *p* < 0.05000, N = 3 (Casewise Deletion of Missing Data)				
	BJ	SK-MEL-3	A375	TF	TP
BJ	R^2^ = 1.0000	R^2^ = −0.9106	R^2^ = 0.4730	R^2^ = −0.7764	R^2^ = −0.7430
	*p* = ---	*p* = 0.271	*p* = 0.686	*p* = 0.434	*p* = 0.467
SK-MEL-3	R^2^ = −0.9106	R^2^ = 1.0000	R^2^ = −0.0665	R^2^ = 0.9675	R^2^ = 0.9532
	*p* = 0.271	*p* = ---	*p* = 0.958	*p* = 0.163	*p* = 0.196
A375	R^2^ = 0.4730	R^2^ = −0.0665	R^2^ = 1.0000	R^2^ = 0.1880	R^2^ = 0.2383
	*p* = 0.686	*p* = 0.958	*p* = ---	*p* = 0.880	*p* = 0.847
TF	R^2^ = −0.7764	R^2^ = 0.9675	R^2^ = 0.1880	R^2^ = 1.0000	**R^2^ = 0.9987**
	*p* = 0.434	*p* = 0.163	*p* = 0.880	*p* = ---	***p* = 0.033**
TP	R^2^ = −0.7430	R^2^ = 0.9532	R^2^ = 0.2383	**R^2^ = 0.9987**	R^2^ = 1.0000
	*p* = 0.467	*p* = 0.196	*p* = 0.847	***p* = 0.033**	*p* = ---

**Red color** indicates selected propolis samples for further biological studies. Units acronyms: P—Pearson’s correlation parameter; R^2^—regression parameter. Cell lines and tests acronyms: BJ—normal human fibroblast cell line; A375—primary malignant melanoma cell line; SK-MEL—highly invasive melanomas originated from lymph cell line; TF—flavonoid content; TP—total polyphenol content.

## Data Availability

The datasets generated during and/or analyzed during the current study are available in the Mendeley Data repository, doi: 10.17632/mdxdntzfwm.1.

## Supplementary Materials

The following supporting information can be downloaded at: https://www.mdpi.com/article/10.3390/molecules31213841/s1, Table S1: Identification of components in propolis extracts by uHPLC-UV-ESI-qTOF-MS/MS; Table S2: Relative abundance of components in propolis extracts; Table S3: Data input for correlation tests of general composition (TP, TF) and activities (DPPH, FRAP, IC50); Table S4: Data input for correlation test of general composition (TP, TF) and caspase activity.
