# Supplementary material for: Targeted Screening Studies on Endemic Propolis Extracts for Selective Cytotoxicity Against Human Melanoma Cells Models
Source: Molecules. 2026 Oct 24;31(21):3841. doi: 10.3390/molecules31213841 (PMC13765220; doi:10.3390/molecules31213841)
Supplement: Supplementary file 1 [file molecules-31-03841-s001.zip › molecules-4580850-supplementary.pdf]

Supplementary Data for “Targeted Screening Studies on Endemic Propolis Extracts for Selective Cytotoxicity Against Human Melanoma Cells Models” by Marta Trzaskowska, Jarosław Widelski, Piotr Okińczyc, Paulina Kazimierczak, Vladyslav Vivcharenko, Anna Bozhadze, Malkhaz Jokhadze, and Agata Przekora.

Table S1. Identification of components in propolis extracts by uHPLC-UV-ESI-qTOF-MS/MS.

No.	Component	RT MS	UV max [nm]	$[M-H]^-$	Base MS/MS Peak	Secondary peaks m/z (A [%])	[M-H+]- Formula	error [mDa]	error [ppm]	RDB
1	Unidentified	0.86	ND/-	179.0565	-	-	$C_6H_{11}O_6$	-0.4	-2.2	1
2	Unidentified	0.86	ND/-	341.1085	-	-	$C_{12}H_{21}O_{11}$	-0.2	-0.4	2
3	Unidentified	0.90	ND/-	133.0144	-	-	$C_4H_5O_5$	-0.2	-1.2	2.0
4	Unidentified	1.32	ND/-	117.0189	-	-	$C_4H_5O_4$	0.3	3.8	2.0
5	[A] 4-Hydroxybenzoic acid	6.45	313, **282**	137.0246	-	-	$C_7H_5O_3$	-0.1	-1.1	5.0
6	[A] Vanillin isomer	9.34	**310, 280**	151.0393	108.2066	-	$C_8H_7O_3$	0.8	5.2	5.0
7	Unidentified	9.74	ND/-	121.0293	-	-	$C_7H_5O_2$	0.2	1.7	5.0
8	[C] Caffeoylglycerol	10.53	***320**	253.0717	133.1749	161.1 (75.43)	$C_{12}H_{13}O_6$	0.1	0.2	6.0
9	[A] Caffeic acid	11.35	**323**	179.0346	135.0449	107.0 (8)	$C_9H_7O_4$	0.4	2.0	6.0
10	[B] Caffeoylglycerol	12.93	**320**	253.0711	161.0743	133.2 (92.59), 135.1 (40.05)	$C_{12}H_{13}O_6$	0.6	2.5	6.0
11	[B] Caffeic acid dihydroxypentyl or isopentyl ester isomer I	14.29	**320**	281.1036	161.1260	133.7 (76.42)	$C_{14}H_{17}O_6$	-0.5	-1.9	6.0
12	[A] *p*-Coumaric acid [a,b,c]	14.36	**310**	163.0401	119.1668	93.1 (10.59)	$C_9H_7O_3$	0.0	-0.1	6.0
13	[C] 3,4,5-Trimethoxy-cinnamic acid	14.39	ND/-	237.0770	145.1304	117.1 (97.17)	$C_{12}H_{13}O_5$	-0.2	-0.8	6.0
14	[B] Caffeic acid dihydroxypentyl or isopentyl ester isomer II	14.71	**320**	281.1034	161.1323	133.1 (55.05), 135.1 (28.11)	$C_{14}H_{17}O_6$	-0.3	-1.0	6.0
15	[A] Ferulic acid	15.16	**325**	193.0504	134.1169	-	$C_{10}H_9O_4$	0.2	1.2	6.0
16	[B] Caffeic acid	15.16	**320**	281.1033	161.1496	133.3 (51.72),	$C_{14}H_{17}O_6$	-0.3	-1.0	6.0

	dihydroxypentyl or isopentyl ester isomer III					135.1 (42.76), 179.1 (10.81)				
17	[B] Plathymenin	15.48	**280**	287.0565	135.1223	151.0 (22.48), 107.2 (12.00), 123.2 (10.12)	$C_{15}H_{11}O_6$	-0.4	-1.3	10.0
18	Unidentified	15.54	315**, 280**	317.0668	284.2400	177.1 (52.34), 200.1 (14.56), 135.3 (15.45), 256.1 (11.30), 228.2 (10.26), 299.3 (9.98), 240.3 (9.16), 124.1 (6.55), 212.2 (4.31), 211.7 (7.91), 215.2 (3.43), 272.2 (2.91)	$C_{16}H_{13}O_7$	-0.1	-0.4	10.0
19	[A] Isoferulic	15.66	**324**	193.0503	134.1466	-	$C_{10}H_9O_4$	0.0	-0.2	6.0
20	[B] Caffeoylmalic acid (Phaseolic acid) isomer	16.64	**334**	295.0827	161.1286	133.3 (58.51), 135.2 (32.02)	$C_{14}H_{15}O_7$	-0.3	-1.1	7.0
21	Unidentified	17.49	***307**	299.0560	240.2314	284.1 (82.87), 228.1 (39.03), 256.1 (34.51), 212.2 (21.86), 200.1 (19.41), 148.2 (11.56), 176.2 (9.58)	$C_{16}H_{11}O_6$	0.1	0.3	11.0
22	Unidentified	17.55	**281**	241.0873	119.0863	197.3 (79.36), 193.2 (17.00)	$C_{15}H_{13}O_3$	-0.2	-1.0	10.0
23	[C] Vanilloyl-methyl-ketone	17.75	*286	193.0505	133.1880	-	$C_{10}H_9O_4$	0.1	0.2	6.0
24	Unidentified	18.29	**280**	301.0725	135.1354	151.1 (18.79), 109.1 (3.20), 137.1	$C_{16}H_{13}O_6$	-0.8	-2.5	10.0

						(3.22), 255.4 (2.92),				
25	[D] *p*-Coumaric acid derivative	18.49	#312	359.1137	145.1441	119.1 (55.67), 163.2 (40.34), 117.3 (26.02), 153.2 (9.79), 150.2 (5.81), 165.2 (5.36)	$C_{19}H_{19}O_7$	0.0	-0.1	10.0
26	[C] Eriodyctiol (4′-hydroxynaringenin)	18.71	**282**	287.0562	125.0569	177.2 (70.51), 201.2 (12.51), 259.2 (12.01), 213.2 (9.60), 241.3 (8.96), 131.2 (7.78)	$C_{15}H_{11}O_6$	-0.1	-0.4	10.0
27	[B] Butin	18.75	**280**, 313	271.0614	135.1276	-	$C_{15}H_{11}O_5$	-0.2	-0.7	10.0
28	Unidentified	19.16	ND/-	273.0763	167.1711	121.3 (52.85), 165.2 (41.06)	$C_{15}H_{13}O_5$	0.6	2.1	9.0
29	Unidentified	19.37	**280**, 342	301.0719	268.2025	177.1 (47.64), 224.2 (37.22), 135.1 (25.85), 283.4 (21.54), 240.2 (15.25), 151.2 (8.34), 196.3 (8.59), 149.2 (7.61), 109.2 (6.69)	$C_{16}H_{13}O_6$	-0.1	-0.5	10.0
30	[B] Caffeic acid dimethyl ether (DMCA)	19.40	**324**	207.0661	-	-	$C_{11}H_{11}O_4$	0.3	1.2	6.0
31	Unidentified	19.42	ND/-	193.0869	147.1921	-	$C_{11}H_{13}O_3$	0.7	3.4	5.0
32	[B] 6,7-dihydroxyflavanon	19.47	**~280**	271.0609	151.1053	107.1 (75.19), 123.2 (65.27), 125.1 (39.60), 95.2 (24.56), 119.2	$C_{15}H_{11}O_5$	0.3	1.2	10.0

						(15.66), 253.2 (13.80), 225.2 (10.23), 185.3 (4.43)				
33	Unidentified	19.52	#**315**	279.0875	145.1378	117.1 (61.44), 119.1 (23.20)	$C_{14}H_{15}O_6$	-0.1	-0.5	7.0
34	Unidentified	20.77	ND	299.0559	284.1704	240.4 (44.39), 148.1 (24.96), 256.2 (15.93), 267.2 (16.54), 211.3 (16.30), 228.1 (8.42), 176.2 (4.96)	$C_{16}H_{11}O_6$	0.3	0.9	11.0
35	[B] Apigenin-7-O-glucoside (Apigetrin)	20.97	309, **265**	431.0983	268.2682	431.3 (23.37), 240.1 (9.85), 211.2 (9.64)	$C_{21}H_{19}O_{10}$	0.0	0.1	12.0
36	Unidentified	21.21	ND/-	263.0921	133.6382	161.1 (74.66)	$C_{14}H_{15}O_5$	0.4	1.7	7.0
37	Unidentified	21.46	ND/-	285.0766	138.1680	224.2 (68.54), 239.3 (45.37)	$C_{16}H_{13}O_5$	-0.1	-0.4	10.0
38	[B] Caffeic ethyl ester	22.45	***320**	207.0664	133.2876	135.1 (48.56), 161.1 (19.75)	$C_{11}H_{11}O_4$	-0.2	-0.8	6.0
39	[B] Liquiritigenin	22.95	**278**, 313	255.0665	119.1291	135.1 (12.99)	$C_{15}H_{11}O_4$	-0.2	-0.8	10.0
40	[C] Aromadendrin (Dihydrokaempferol)	23.01	288	287.0560	135.1477	-	$C_{15}H_{11}O_6$	0.1	0.4	10.0
41	[B] Pinobanksin-5-methylether	23.19	**287**	285.0777	252.0429	224.0 (55.83), 138.0 (38.07), 241.0 (31.50), 165.0 (14.95), 239.1 (12.13), 195.0 (12.02), 151.0 (11.81), 213.1 (11.34), 267.1 (11.02),	$C_{16}H_{13}O_5$	-0.8	-2.9	10.0

42	Unidentified	23.65	**281**, 300sh	285.0770	135.0957	285.1 (9.31), 136.0 (8.53), 107.0 (6.81) 270.2 (4.98), 91.2 (2.82)	$C_{16}H_{13}O_5$	-0.2	-0.7	10.0
43	Unidentified	23.81	**216**, 283	255.0668	209.2021	237.1 (66.77), 135.1 (35.83), 181.2 (18.90), 109.1 (14.80), 193.2 (13.54), 149.1 (11.31), 255.2 (10.78), 199.3 (11.34), 165.2 (8.91), 197.3 (6.94), 141.3 (6.36), 195.2 (5.68), 224.3 (5.95), 167.3 (5.45)	$C_{15}H_{11}O_4$	-0.5	-2.0	10.0
44	[D] Caffeic acid derivate	23.87	**326**	277.1088	161.1124	135.1 (24.48), 277.3 (13.68), 137.2 (7.87)	$C_{15}H_{17}O_5$	0.0	-0.1	7.0
45	[B] 2'-Hydroxyformononetin	23.87	ND	283.0616	268.2094	211.3 (38.75), 240.2 (19.50), 135.1 (9.95), 184.2 (10.15), 148.1 (9.07), 224.2 (5.35), 196.2 (3.41), 251.2 (2.17)	$C_{16}H_{11}O_5$	-0.4	-1.5	11.0
46	Unidentified	24.32	ND/-	329.0673	299.2387	314.2 (53.64), 271.1 (48.64), 243.1 (14.44), 329.	$C_{17}H_{13}O_7$	-0.9	-2.9	11.0

						3 (6.82)				
47	[B] Odoratin	24.83	**260**, 320	313.0719	255.2335	283.2 (83.04), 298.2 (54.94), 211.1 (14.26), 270.2 (10.80)	$C_{17}H_{13}O_6$	-0.1	-0.4	11.0
48	[A] Quercetin	24.93	364, 270sh, **265**	301.0353	151.0034	121.0 (29.41), 107.0 (22.18), 149.0 (14.01), 178.9 (13.92), 301.0 (7.58), 245.0 (6.32), 273.0 (5.48), 163.0 (4.87), 211.0 (3.84)	$C_{15}H_9O_7$	0.1	0.3	11.0
49	[A] Luteolin	25.29	***370**	285.0412	133.1356	285.2 (83.77), 151.0 (33.21), 199.1 (15.09), 107.1 (12.83)	$C_{15}H_9O_6$	-0.8	-2.7	11.0
50	Unidentified	25.32	ND	273.0769	151.1730	109.1 (31.37)	$C_{15}H_{13}O_5$	0.0	0.0	9.0
51	Unidentified	25.44	ND	229.0510	159.1509	-	$C_{13}H_9O_4$	-0.4	-1.6	9.0
52	Unidentified	26.58	**292**	301.0723	166.1341	110.1 (43.48), 255.3 (10.26)	$C_{16}H_{13}O_6$	-0.5	-1.7	10.0
53	[B] Quercetin-3-methyl ether	26.70	355, 268sh, **255**	315.0497	271.0253	300.0 (71.14), 255.0 (42.89) 243.0 (22.59), 227.0 (2.55)	$C_{16}H_{11}O_7$	0.2	0.5	11.0
54	[A] Pinobanksin	26.99	**292**	271.0615	197.0617	253.0 (89.28), 161.1 (67.51), 271.1 (56.26), 125.0 (53.39), 151.0 (30.14), 225.1 (24.71), 107.0 (23.97),	$C_{15}H_{11}O_5$	-0.3	-1.1	10.0

						209.1 (16.07), 185.1 (15.86), 115.1 (15.08), 157.1 (14.43), 181.1 (14.14), 215.1 (11.83)				
55	Unidentified	27.97	ND	255.0668	107.1467	213.2 (93.61), 151.0 (89.35), 124.1 (52.21), 185.2 (34.78), 157.1 (19.16), 164.1 (18.89), 227.3 (7.85), 193.3 (7.41)	$C_{15}H_{11}O_4$	-0.5	-2.0	10.0
56	[A] Naringenin	28.40	**282**	271.0612	119.1344	151.0 (43.37), 107.1 (21.94), 187.2 (10.00)	$C_{15}H_{11}O_5$	0.0	0.1	10.0
57	[B] Chrysin-5-methyl-ether	28.78	-	267.0662	224.1747	180.2 (92.97), 252.2 (26.27), 195.3 (15.00)	$C_{16}H_{11}O_4$	0.1	0.3	11.0
58	Unidentified	28.89	**371**	271.0615	135.1477	-	$C_{15}H_{11}O_5$	-0.3	-1.0	10.0
59	Unidentified	29.35	***282**	269.0822	150.0692	184.2 (88.87), 165.1 (80.74), 122.1 (55.22), 254.2 (50.90), 227.2 (38.24), 269.3 (20.13)	$C_{16}H_{13}O_4$	-0.3	-1.0	10.0
60	[C] Naringenin chalcone	30.09	**290**	271.0621	125.0849	253.2017 (15.19), 197.2178 (14.62)	$C_{15}H_{11}O_5$	-0.6	-2.0	10.0
61	[C] Aromadendrin-7-methyl ether	30.13	**287**	301.0717	151.0669	134.1 (98.98), 164.1 (57.48), 149.2 (51.43), 136.1 (36.27),	$C_{16}H_{13}O_6$	0.3	1.1	10.0

						108.2 (29.30)				
62	[A] Apigenin	30.41	**338**, 290sh, 263	269.0457	117.0349	269.0 (52.06), 151.0 (39.01), 149.0 (25.91), 227.0 (12.66), 107.0 (11.48), 225.0 (10.59), 201.1 (7.44), 183.0 (6.40), 181.1 (5.14), 121.0 (4.92), 197.1 (2.28)	$C_{15}H_9O_5$	-0.2	-0.7	11.0
63	[A] Kaempferol	31.01	**366**, 295sh, 265	285.0405	285.0400	239.0 (8.81), 187.0 (8.20), 185.1 (8.14), 229.0 (7.99), 159.0 (6.63)	$C_{15}H_9O_6$	-0.1	-0.3	11.0
64	[B] Caffeic acid hydroxyphenylethyl ester	31.26	***324**	299.0925	135.0841	179.1 (25.83), 161.2 (7.79)	$C_{17}H_{15}O_5$	0.7	2.3	10.0
65	[A] Quercetin-3′-methyl ether (Isorhamnetin)	31.58	***253**, 357	315.0509	300.1989	151.1 (26.66), 271.4 (11.37), 164.1 (7.61), 283.1 (6.12), 148.1 (5.64), 315.2 (5.60), 255.2 (4.65), 216.2 (3.38), 108.2 (2.95), 244.2 (2.60), 136.2 (2.55)	$C_{16}H_{11}O_7$	0.1	0.3	11.0
66	Unidentified	31.94	**280**, 312	271.0615	151.0766	135.2 (13.44)	$C_{15}H_{11}O_5$	-0.1	-0.3	10.0
67	[B] Quercetin-methyl-ether	32.12	-	315.0511	300.1857	151.1 (26.12),	$C_{16}H_{11}O_7$	0.0	-0.1	11.0

						271.3 (11.15), 164.1 (7.58), 283.1 (5.81), 216.3 (4.63)				
68	[B] Luteolin-5-methyl ether	32.84	350, 298sh, **267**	299.0549	255.0300	227.0 (59.96), 284.0 (15.07), 211.0 (6.11)	$C_{16}H_{11}O_6$	-0.2	-0.7	11.0
69	[B] Quercetin-di-methyl-ether isomer I	33.56	256, 354	329.0669	271.1688	299.2 (99.34), 243.2 (90.63), 285.4 (51.12), 257.2 (31.51), 314.2 (29.44), 227.2 (5.23), 215.2 (3.74), 199.2 (3.06), 255.1 (2.88)	$C_{17}H_{13}O_7$	-0.2	-0.6	11.0
70	[B] Caffeic acid butenyl or isobutenyl ester	33.60	ND/-	233.0830	133.3638	-	$C_{13}H_{13}O_4$	-1.1	-4.7	7.0
71	[B] Dalbergin	33.79	**249**, 302	267.0675	252.2179	223.4 (46.13), 195.2 (18.44), 132.1 (12.50), 135.1 (8.21), 208.2 (7.21), 267.3 (2.60)	$C_{16}H_{11}O_4$	-1.0	-3.6	11.0
72	Unidentified	34.04	**294**	253.0879	237.1859	161.1 (35.58), 136.2 (14.89), 210.2 (9.94), 193.2 (7.93)	$C_{16}H_{13}O_3$	-0.9	-1.5	10.0
73	[B] 1,3-di-*p*-Coumaroylglycerol	33.80	**312**	383.1143	163.1491	119.1 (69.49), 145.1 (61.09), 117.2 (8.68), 219.2 (7.20), 237.2 (6.59), 383.4	$C_{21}H_{19}O_7$	-0.7	-1.8	12.0

						(2.42)				
74	B Galangin-5-methyl-ether	34.27	**353**	283.0612	211.1796	239.2 (58.94), 283.3 (5.07), 268.2 (4.79)	$C_{16}H_{11}O_5$	0.0	-0.1	11.0
75	B 5-Methyl-Pinobanksin--3- acetate	34.35	**280**	327.0878	224.1781	267.2 (67.46), 252.2 (62.85), 285.2 (45.11), 239.5 (36.67)	$C_{18}H_{15}O_6$	-0.4	-1.1	11.0
76	B 7-Hydroxyflavanone	34.66	**278**, 313	239.0724	197.1860	135.1 (75.29), 169.2 (19.30), 239.2 (12.20), 141.1 (8.12), 148.2 (8.64), 91.4 (10.95), 109.1 (4.75), 153.2 (4.66), 167.4 (4.79), 153.2 (2.32), 152.7 (4.09)	$C_{15}H_{11}O_3$	-1.0	-4.1	10.0
77	B 2-Acetyl-1,3-di-caffeoylglycerol	35.15	**320**	457.1141	179.1565	161.1 (77.42), 135.1 (45.90), 235.2 (48.11), 295.3 (38.65), 457.3 (5.86), 173.2 (3.85), 397.4 (4.20), 413.6 (3.26), 253.2 (2.22)	$C_{23}H_{21}O_{10}$	-0.1	-0.2	13.0
78	Unidentified	35.25	280, 313	271.0983	135.1226	241.1 (40.12), 109.2 (37.44), 121.3 (42.86), 197.2 (36.10), 149.2 (32.53),	$C_{16}H_{15}O_4$	-0.7	-2.5	9.0

79	B Isoliquiritigenin	35.38	**282,** 373	255.0671	119.1139	214.2 (15.90), 227.2 (11.38), 186.7 (15.40) 135.1 (11.69) 121.1 (39.04),	$C_{15}H_{11}O_4$	-0.8	-3.0	10.0
80	A Quercetin-7-methyl ether (Rhamnetin)	36.29	**362**	315.0509	165.1079	300.2 (27.72), 151.1 (9.49), 272.2 (6.69), 244.2 (4.72), 256.3 (3.45)	$C_{16}H_{11}O_7$	0.1	0.4	11.0
81	B Kaempferol-methyl-ether	36.44	ND/-	299.0563	284.1907	299.2 (7.35), 256.1 (5.21), 133.2 (5.23), 151.1 (2.37), 227.3 (2.53)	$C_{16}H_{11}O_6$	-0.2	-0.7	11.0
82	B Caffeic acid butyl or isobutyl ester isomer I	36.91	**325**	235.0976	161.1424	135.1 (93.59)	$C_{13}H_{15}O_4$	-0.1	-0.2	6.0
83	B Pinobanksin-3-O-hydroxybutyrate or isobutyrate	37.24	**278**	357.0975	253.2301	271.3 (5.41), 197.2 (4.82)	$C_{19}H_{17}O_7$	0.5	1.3	11.0
84	B Caffeic acid prenyl ester isomer	37.41	**320**	247.0975	135.1279	161.1 (33.38)	$C_{14}H_{15}O_4$	0.0	0.2	7.0
85	B Caffeic acid butyl or isobutyl ester isomer II	38.15	**320**	235.0976	161.1424	135.1 (93.59)	$C_{13}H_{15}O_4$	–0.1	–0.2	6.0
86	B Quercetin-di-methyl-ether isomer II	38.55	**353**	329.0669	299.1970	271.2 (30.28), 314.2 (21.06), 285.2 (2.46)	$C_{17}H_{13}O_7$	-0.3	-0.8	11.0
87	B Caffeic acid 2-methyl-2-butenyl ester	38.74	**325**	247.0979	135.1258	161.1 (36.02), 179.1 (11.25)	$C_{14}H_{15}O_4$	-0.4	-1.5	7.0
88	B Medicarpin	39.09	**286**	269.0826	254.2278	211.4 (30.12), 239.1 (19.70), 185.4 (19.26), 226.2 (12.70),	$C_{16}H_{13}O_4$	-0.6	-2.4	10.0

						237.2 (7.12), 197.9 (7.39)				
89	[B] Caffeic acid 3-methyl-2-butenyl ester (Basic prenyl ester)	40.14	**325**	247.0979	134.2235	106.1 (6.32)	$C_{14}H_{15}O_4$	-0.4	-1.7	7.0
90	[B] Caffeic acid 3-methyl-3-butenyl ester	40.60	**325**	247.0977	134.2234	106.2 (5.64)	$C_{14}H_{15}O_4$	-0.1	-0.4	7.0
91	[B] 2-Acetyl-1-caffeoyl-3-p-coumaroylglycerol	41.48	**315**	441.1197	163.1479	179.1 (85.75), 161.1 (42.10), 135.1 (40.85), 145.2 (39.56), 119.1 (35.73), 235.2 (27.59), 295.3 (14.64), 219.2 (7.31), 173.2 (6.88), 381.4 (7.79), 217.2 (4.50), 441.3 (4.75), 189.2 (3.80), 277.3 (2.86)	$C_{23}H_{21}O_9$	-0.6	-1.3	13.0
92	[C] Methylated flavonoid	41.59	***281**	253.0879	237.2078	161.1 (27.57), 209.5 (30.58), 136.2 (15.98), 108.1 (11.54), 120.2 (7.98)	$C_{16}H_{13}O_3$	-0.8	-3.3	10.0
93	[A] Chrysin	41.80	**268,** 312sh	253.0505	253.0507	143.0 (41.53), 145.0 (21.10), 209.1 (14.10), 107.0 (13.33), 181.1 (8.16), 185.1 (6.19)	$C_{15}H_9O_4$	-0.7	-2.8	11.0
94	[B] Caffeic acid benzyl	41.95	**326**	269.0818	134.1302	161.0 (22.96),	$C_{16}H_{13}O_4$	-0.3	-1.1	10.0

	ester					137.0 (4.03)				
95	A Pinocembrin	42.64	**290**	255.0666	171.0464	151.0 (80.69), 255.1 (75.17), 213.1 (74.89), 145.1 (70.09), 107.0 (52.59), 185.1 (34.69), 169.1 (24.91), 211.1 (23.68), 164.0 (17.93), 187.1 (16.78), 136.0 (16.34)	$C_{15}H_{11}O_4$	-0.2	-0.8	10.0
96	A Pinocembrin chalcone	42.90	**341**	255.0668	171.2496	151.1 (56.63), 107.2 (32.90), 145.2 (23.49), 211.2 (16.51), 169.2 (15.88), 255.3 (12.61), 141.2 (8.45), 213.2 (8.89), 183.2 (7.08), 187.2 (5.71), 133.2 (3.21)	$C_{15}H_{11}O_4$	-0.5	-1.9	10.0
97	A Naringenin-4′-methyl ether (Isosakuranetin)	43.77	**290**	285.0773	124.1060	139.1 (64.17), 145.1 (42.28), 148.1 (8.73), 165.1 (4.71)	$C_{16}H_{13}O_5$	-0.4	-1.6	10.0
98	B Ermanin isomer	43.98	333, **275**	313.0721	283.1860	298.2 (15.38), 255.2 (14.54), 163.1 (7.37), 227.1 (3.56), 117.1 (2.59), 165.2 (3.29)	$C_{17}H_{13}O_6$	-0.3	-1.1	11.0

99	A Galangina	44.42	360, **266**	269.0454	269.0454	169.1 (12.64), 171.0 (10.87), 213.0 (10.73), 143.0 (8.90), 223.0 (8.03,) 195.0 (7.34)	$C_{15}H_9O_5$	-0.2	-0.8	11.0
100	B 4-Metoxydalbergione	44.91	**263**	255.1029	136.1210	108.1 (62.20), 149.1 (28.79)	$C_{16}H_{15}O_3$	-0.2	-0.8	9.0
101	A Pinocembrin dihydrochalcone	45.01	**art**	257.0826	213.2258	173.2 (69.29), 171.2 (39.44), 151.1 (32.96), 122.1 (20.68), 156.2 (21.79), 195.2 (14.59), 257.2 (13.28), 239.3 (12.91), 169.2 (11.80), 147.3 (7.77)	$C_{15}H_{13}O_4$	-0.7	-2.6	9.0
102	A Apigenin-3′-methyl ether (Acacetin) or A Apigenin-7-methyl ether (Genkwanin)	45.14	335, **269**	283.0619	268.2009	240.2 (6.84), 151.1 (4.26), 283.3 (2.19)	$C_{16}H_{11}O_5$	-0.7	-2.3	11.0
103	B Caffeic acid pentyl or isopentylester	46.00	-	249.1138	161.1050	-	$C_{14}H_{17}O_4$	-0.6	-2.3	6.0
104	A Caffeic acid phenethyl ester (CAPE)	46.30	326	283.0981	135.1231	161.1 (46.24), 179.1 (20.40)	$C_{17}H_{15}O_4$	-0.6	-2.0	10.0
105	B Pinobanksin-3-acetate	46.40	**295**	313.0725	253.0510	197.1 (5.86), 271.1 (5.36), 209.1 (4.75), 143.0 (3.17)	$C_{17}H_{13}O_6$	-0.7	-2.3	16.0
106	A Kaempferide (Kaempferol-4'-methyl ether)	46.80	**365**, 267	299.0563	284.2046	151.1 (31.84), 164.1 (10.53), 107.2 (6.32), 132.1	$C_{16}H_{11}O_6$	-0.2	-0.7	11.0

						(4.91), 228.2 (3.34), 299.2 (3.46), 200.2 (2.10), 256.1 (2.02)				
107	B Methoxychrysin	47.23	**266,** 310sh	283.0614	211.0405	239.0 (65.55), 268.0 (8.80)	$C_{16}H_{11}O_5$	-0.2	-0.6	11.0
108	B Ermanin (Kaempferol-3,4′-dimethyeter kemferolu)	49.46	**350**, 267	313.0719	283.2122	255.2 (24.32), 253.2 (17.11), 298.2 (10.64)	$C_{17}H_{13}O_6$	-0.1	-0.3	11.0
109	B *p*-Coumaric acid 3-methyl-3-butenyl ester	49.69	**313**	231.1028	117.1725	119.1 (90.59), 145.1 (49.02), 163.1 (4.99)	$C_{14}H_{15}O_3$	-0.1	-0.4	7.0
110	B 2-Acetyl-1,3-di-*p*-coumaroylglycerol	50.39	**312**	425.1242	163.0403	145.0 (53.67), 119.0 (49.02), 219.1 (11.88), 215.1 (6.36), 237.1 (5.21), 171.1 (5.05), 117.0 (4.31)	$C_{23}H_{21}O_8$	0.0	0.1	13.0
111	B 2-Acetyl-3-*p*-coumaroyl-1-feruloylglycerol	51.06	**316**	455.1336	163.1189	193.2 (95.43), 134.1 (43.39), 119.1 (41.07), 145.1 (38.25), 175.4 (43.52), 160.7 (15.25)	$C_{24}H_{23}O_9$	1.1	2.5	13.0
112	B *p*-Coumaric acid 3-methyl-2-butenyl or 2-methyl-2-butenyl	51.18	**313**	231.1027	117.2347	-	$C_{14}H_{15}O_3$	0.0	0.0	7.0
113	Unidentified	51.35	**276,** 310sh	241.0870	122.1167	150.1 (99.27), 135.2 (52.25), 109.1 (35.93)	$C_{15}H_{13}O_3$	0.0	0.1	9.0
114	B 1-Acetyl-2,3-di-*p*-	51.67	**311**	425.1244	163.1361	145.1 (64.46),	$C_{23}H_{21}O_8$	-0.2	-0.4	13.0

	coumaroylglycerol					119.1 (57.20), 219.2 (13.02), 171.3 (7.70)				
115	[B] 2-Acetyl-1,3-di-feruloylglycerol	51.90	**324**	485.1456	193.1733	175.1 (33.53), 134.1 (31.79), 149.2 (12.96), 249.2 (8.24), 230.3 (7.88), 160.3 (7.78), 425.4 (4.94), 207.1 (4.01), 470.4 (4.63)	$C_{25}H_{25}O_{10}$	-0.3	-0.5	13.0
116	Unidentified	52.67	ND/-	373.2021	373.4762	173.3 (90.08), 217.3 (53.54), 137.1 (34.39)	$C_{22}H_{29}O_5$	0.0	0.0	8.0
117	[B] *p*-Coumaric acid benzyl ester	52.93	**316**	253.0869	117.2666	145.1 (12.89), 121.3 (3.15)	$C_{16}H_{13}O_3$	0.1	0.3	10.0
118	Unidentified	53.54	**280**	239.0721	135.0585	197.3 (36.47), 239.2 (22.75), 169.4 (14.89), 193.3 (7.87)	$C_{15}H_{11}O_3$	-0.7	-2.9	10.0
119	Unidentified	54.27	**305**	433.0927	243.2176	271.2 (40.28), 415.4 (25.86), 161.1 (21.48), 253.2 (10.71), 125.1 (7.37), 135.1 (6.47), 165.1 (5.55), 152.1 (5.35), 180.1 (4.98), 227.2 (4.58), 199.3 (4.10), 371.3 (3.52), 280.2	$C_{24}H_{17}O_8$	0.2	0.4	16.0

						(2.60)				
120	[B] Ferulic acid benzyl ester	54.53	**320**	283.0975	133.1109	160.2 (13.55)	$C_{17}H_{15}O_4$	0.1	0.4	10.0
121	[B] Caffeic acid cinnamyl ester	55.23	**323**	295.0982	134.1352	161.1 (5.53), 137.1 (5.18), 106.1 (4.21)	$C_{18}H_{15}O_4$	-0.6	-1.9	11.0
122	[B] Pinobanksin-3-propanoate	57.35	**294**	327.0878	253.2179	197.2 (5.41), 209.2 (3.72), 271.3 (2.71), 143.2 (2.09)	$C_{18}H_{15}O_6$	-0.4	-1.2	11.0
123	[B] Caffeic acid hexyl or isohexyl ester	57.50	ND/-	263.1297	161.1162	135.1 (75.18)	$C_{15}H_{19}O_4$	-0.8	-3.2	6.0
124	[B] *p*-Coumaric acid phenethyl ester	57.64	**310**	267.1031	119.1219	145.1 (81.97), 117.2 (80.24), 163.1 (11.83)	$C_{17}H_{15}O_3$	-0.4	-1.6	10.0
125	[B] Pinostrobin chalcone	59.79	**343**	269.0827	122.0703	165.1 (83.49), 253.4 (86.88), 177.2 (49.29), 226.2 (47.58), 171.1 (35.51), 150.1 (31.31), 163.1 (21.30), 269.2 (16.42), 136.1 (13.47), 198.2 (14.25)	$C_{16}H_{13}O_4$	-0.3	-0.8	10.0
126	[C] 2′,6′-Dihydroxy−4′,4-dimethoxy dihydrochalcone (Calomelanone)	61.46	**285**	271.0979	152.0937	124.1 (60.13), 210.2 (27.77), 238.3 (25.34), 173.2 (13.05), 165.1 (10.13), 271.2 (7.97), 253.2 (6.31)	$C_{16}H_{15}O_4$	−0.3	−1.1	9.0
127	[A] Tectochrysin (Chrysin-	61.90	313, **268**	269.0815	269.2764	226.2 (59.49),	$C_{16}H_{13}O_4$	−0.7	−2.5	11

	7-methyl ether) [M+H]!					254.2 (23.65), 167.1 (8.30), 270.5 (6.16), 186.3 (4.73), 129.1 (2.37), 209.2 (2.14)				
128	[A] Pinostrobin (Pinocembrin-7-methyl ether) [M+H]!	62.40	**288**	271.0969	167.1288	131.1 (33.29), 103.2 (24.27), 269.3 (11.76), 226.3 (8.78), 271.2 (4.49), 270.5 (3.31), 254.3 (2.97), 186.3 (2.31), 165.2 (2.29)	$C_{16}H_{15}O_4$	−0.4	−1.5	10
129	[B] Pinobanksin-3-butenoate or isobutenoate	62.60	ND/-	339.0862	253.2304	197.2058 (5.17)	$C_{19}H_{15}O_6$	-0.9	-2.8	12
130	[B] *p*-Coumaric acid cinnamyl ester	63.68	**313**	279.1029	117.3253	-	$C_{18}H_{15}O_3$	-0.3	-1.0	11.0
131	[B] Pinobanksin-3-butanoate or isobutanoate	64.47	**293**	341.1037	253.2173	197.2 (4.89), 209.2 (3.17)	$C_{19}H_{17}O_6$	-0.6	-1.8	11.0
132	Unidentified	64.88	ND	295.2286	-	-	$C_{18}H_{31}O_3$	-0.7	-2.3	3.0
133	[B] Pinobanksin-3-pentenoate or isopentenoate I	65.16	**292**	353.1039	253.2231	197.2 (4.88), 209.2 (2.96)	$C_{20}H_{17}O_6$	-0.9	-2.5	12.0
134	[B] Pinobanksin-3-pentenoate or isopentenoate II	65.48	**282**	353.1035	253.2266	271.2 (26.83), 197.3 (5.55), 209.6 (3.51), 225.3 (2.59)	$C_{20}H_{17}O_6$	-0.5	-1.9	12.0
135	Unidentified	66.09	ND/-	413.1971	161.1344	134.3 (86.37), 137.3 (17.10),	$C_{24}H_{29}O_6$	-0.2	-0.4	10.0

136	[D] Caffeic acid derivate	66.15	ND/-	399.2204	134.1728	179.2 (16.17), 251.3 (15.45), 415.4 (5.43) 178.3 (37.66), 399.6 (27.29)	$C_{24}H_{31}O_5$	-0.7	-1.7	9.0
137	[B] Pinobanksin-3-benzoate	66.61	***278**	375.0878	253.2202	197.1 (4.84), 225.2 (3.56), 121.2 (3.04), 209.2 (2.85)	$C_{22}H_{15}O_6$	-0.4	-1.0	15.0
138	[B] Pinobanksin-derivate	67.40	**291**	389.1034	253.2174	271.2 (50.52)	$C_{23}H_{17}O_6$	-0.3	-0.9	15.0
139	[B] Pinobanksin-3-pentanoate or isopentenoate I	67.56	**293**	355.1192	253.2167	197.2 (4.62), 271.2 (3.55), 209.2 (2.17)	$C_{20}H_{19}O_6$	-0.5	-1.5	11.0
140	[B] Pinobanksin-3-pentanoate or isopentenoate II	67.70	**293**	355.1194	253.2180	197.2 (4.47), 209.2 (2.52)	$C_{20}H_{19}O_6$	-0.6	-1.8	11.0
141	[C] Caffeic acid monoterpene (geranyl) ester	67.88	**326**	315.1606	134.2110	137.1 (4.72), 179.1 (2.29)	$C_{19}H_{23}O_4$	-0.4	-1.3	8.0
142	Unidentified	67.98	-	463.3284	283.4493	-	$C_{24}H_{47}O_8$	-0.8	-1.7	1.0
143	[B] Pinobanksin-3-hexenoate or isohexenoate	68.36	ND/-	367.1189	253.2181	271.2 (31.89), 197.3 (5.77), 209.5 (3.20), 225.3 (2.91)	$C_{21}H_{19}O_6$	-0.2	-0.4	12.0
144	[B] Pinobanksin-3-hydroxycinnamate	69.05	**285**	403.1197	253.2276	271.2 (4.98), 197.2 (4.05), 225.3 (2.92), 149.1 (2.44)	$C_{24}H_{19}O_6$	-1.0	-2.5	15.0
145	[B] Metoxycinnamic acid cinnamyl ester	69.11	**282**	293.2125	293.4701	185.2 (57.87), 125.2 (49.45), 141.2 (18.74), 197.3 (15.90), 97.2 (11.61)	$C_{18}H_{29}O_3$	-0.3	-0.9	4.0

146	Unidentified	69.39	**293**	521.1755	287.3045	453.5 (54.06), 165.2 (22.47), 475.5 (22.74), 261.3 (5.68)	$C_{36}H_{25}O_4$	0.3	0.6	24.0
147	B Pinobanksin-3-hexanoate or isohexanoate I	69.40	**281**	369.1347	253.2138	271.2 (4.95), 197.2 (3.43), 225.1 (2.37), 115.2 (1.95)	$C_{21}H_{21}O_6$	-0.3	-0.8	11.0
148	Unidentified	69.56	**291**	491.1658	287.3047	445.5 (37.73), 423.5 (28.43), 135.1 (13.25), 261.2 (3.44)	$C_{35}H_{23}O_3$	-0.5	-1.0	24.0
149	Unidentified	69.60	ND/-	357.2082	137.1078	357.4 (66.04), 219.4 (27.83), 93.1 (19.01), 149.3 (8.10)	$C_{22}H_{29}O_4$	-0.7	-1.8	8.0
150	B Metoxycinnamic acid cinnamyl ester isomer	69.66	**282**	293.2132	293.5352	185.3 (57.73)	$C_{18}H_{29}O_3$	-0.9	-3.0	4.0
151	B Pinobanksin-3-hexanoate or isohexanoate II	69.70	**294**	369.1347	253.2245	197.2 (4.52), 271.2 (3.90), 225.3 (2.22), 209.2 (1.98), 115.1717 (1.93)	$C_{21}H_{21}O_6$	-0.3	-0.8	11.0
152	Unidentified	70.10	-	343.2855	283.3972	211.3 (96.37), 197.3 (72.36), 253.4 (30.83), 279.5 (19.71)	$C_{20}H_{39}O_4$	-0.1	-0.3	1.0
153	Unidentified	70.58	-	295.2279	295.4866	141.2 (52.92)	$C_{18}H_{31}O_3$	0.0	-0.1	3.0
154	Pinobanksin-3-phenylpentenoate or phenylisopentenoate ester	70.73	***282**	429.1344	253.2249	271.2 (57.79), 197.2 (3.17), 225.4 (3.81)	$C_{26}H_{21}O_6$	0.0	-0.1	16.0
155	Unidentified	71.08	**291**	535.1929	467.5169	287.2 (10.42),	$C_{25}H_{31}N_2O_{11}$	0.4	0.8	11.0

						179.1 (2.02)				
156	Unidentified	71.19	ND/-	505.3388	283.4757	-	$C_{26}H_{49}O_9$	-0.6	-1.2	2
157	Unidentified	71.29	**293**	407.1852	287.3167	119.1 (37.66), 407.4 (41.34), 201.3 (13.02), 219.2 (9.35), 243.3 (8.66), 283.3 (6.58), 176.4 (7.08), 218.2 (5.25), 313.3 (4.86), 301.4 (4.19), 190.2 (3.08), 259.3 (2.98), 163.4 (3.66), 261.4 (2.63), 199.3 (2.13)	$C_{25}H_{27}O_5$	1.2	2.9	12
158	Unidentified	71.37	-	341.2126	297.4220	341.4 (55.57), 150.1 (49.63), 203.3 (33.41), 107.1 (27.22), 339.5 (27.06), 159.5 (24.45), 183.2 (14.05), 147.2 (5.88), 119.2 (5.82), 189.2 (4.90)	$C_{22}H_{29}O_3$	-0.4	-1.2	8.0
159	Unidentified	71.73	-	491.3597	311.5394	-	$C_{26}H_{51}O_8$	-0.8	-1.5	1.0
160	Unidentified	73.66	-	521.3270	453.6251	521.6 (27.27)	$C_{33}H_{45}O_5$	0.3	0.5	11
161	Unidentified	76.54	-	521.3272	453.6309	521.7 (50.64), 522.7 (2.13)	$C_{33}H_{45}O_5$	0.2	0.1	11
162	Unidentified	76.75	-	521.3270	453.6251	521.6 (27.27)	$C_{33}H_{45}O_5$	0.3	0.5	11

Abbreviations: A(%) – relative abundance; RT – retention time; A – identification by comparison of UV and MS/MS spectra with standards (the highest level of confidence); B – identification by comparison of MS/MS and/or UV spectrum with literature (good level of confidence); C – component was identified according to deprotonated molecular ion formula and prediction from MS/MS spectra detected in *Populus* genus in literature, but there are no sufficient MS and/or UV data (average/weak level of confidence); D – component was identified according to deprotonated molecular ion and prediction from MS spectra, but there are no sufficient MS/MS, and UV data and components have not been reported in poplars in literature (the weakest level of confidence); [M + H]! – components does not produce ions in ESI-negative mode; therefore, positive fragmentation was presented; ND/- – UV maximum was not determined due to low concentration, overlapping peaks or lack of UV absorption by components; * – UV maximum was evaluated approximately due to low concentration or overlapping peaks.

Table S2. Relative abundance of components in propolis extracts

No. RT	Component		NE-TRI	GR-PA	KA-BO	UK-TA	GE-OR	GE-OT	GE-Q1	GE-Q2	GE-VA1	GE-VA2
1	Unidentified	0.86	-	tr	+	tr	+	tr	tr	tr	tr	tr
2	Unidentified	0.86	-	tr	tr	tr	tr	tr	tr	-	tr	tr
3	Unidentified	0.90	-	-	tr	-	tr	tr	tr	-	-	-
4	Unidentified	1.32	-	-	tr	-	-	-	tr	-	-	-
5	[A] 4-Hydroxybenzoic acid	6.45	-	tr	tr	-	tr	tr	tr	tr	tr	-
6	[A] Vanillin isomer	9.34	-	-	tr	-	-	-	tr	-	-	-
7	Unidentified	9.74	-	-	-	tr	-	-	-	-	-	-
8	[C] Caffeoylglycerol	10.53	-	-	tr	-	-	-	-	-	-	-
9	[A] Caffeic acid	11.35	-	+	+	tr	++	+	++	+	tr	tr
10	[B] Caffeoylglycerol	12.93	-	-	tr	tr	tr	tr	tr	tr	tr	tr
11	[B] Caffeic acid dihydroxypentyl or isopentyl ester isomer I	14.29	-	-	-	-	tr	tr	tr	tr	tr	-
12	[A] *p*-Coumaric acid [a,b,c]	14.39	-	-	tr	-	-	-	-	-	-	-
13	[C] 3,4,5-Trimethoxy-cinnamic acid	14.36	-	tr	+	+	++	+	+	+	tr	tr

No.	Component	RT	NE-TRI	GR-PA	KA-BO	UK-TA	GE-OR	GE-OT	GE-Q1	GE-Q2	GE-VA1	GE-VA2
14	[B] Caffeic acid dihydroxypentyl or isopentyl ester isomer II	14.71	-	tr	tr	tr	tr	tr	tr	tr	tr	-
15	[A] Ferulic acid	15.16	-	tr	tr	tr	tr	tr	tr	tr	tr	-
16	[B] Caffeic acid dihydroxypentyl or isopentyl ester isomer III	15.16	-	tr	tr	tr	tr	tr	tr	tr	tr	-
17	[B] Plathymenin	15.48	tr	-	-	-	-	-	-	-	-	-
18	Unidentified	15.54	tr	-	-	-	-	-	-	-	-	-
19	[A] Isoferulic	15.66	-	tr	tr	tr	+	tr	+	tr	tr	-
20	[B] Caffeoylmalic acid (Phaseolic acid) isomer	16.60	-	-	tr	-	tr	-	tr	tr	-	-
21	Unidentified	17.49	tr	-	-	-	-	-	-	-	-	-
22	Unidentified	17.55	tr	-	-	-	-	-	-	-	-	-
23	[C] Vanilloyl-methyl-ketone	17.75	-	-	-	-	tr	-	tr	tr	-	-
24	Unidentified	18.29	tr	-	-	-	-	-	-	-	-	-
25	[D] *p*-Coumaric acid derivative	18.49	-	-	-	tr	-	-	-	-	-	-
26	[C] Eriodyctiol (4'-hydroxynaringenin)	18.71	-	tr	tr	tr	tr	tr	tr	tr	tr	-
27	[B] Butin	18.75	tr	-	-	-	-	-	-	-	-	-
28	Unidentified	19.16	tr	-	-	-	-	-	-	-	-	-
29	Unidentified	19.37	tr	-	-	-	-	-	-	-	-	-
30	[B] Caffeic acid dimethyl ether (DMCA)	19.40	-	tr	tr	-	tr	tr	tr	tr	tr	-
31	Unidentified	19.42	-	-	-	-	tr	tr	tr	-	-	-

No.	Component	RT	NE-TRI	GR-PA	KA-BO	UK-TA	GE-OR	GE-OT	GE-Q1	GE-Q2	GE-VA1	GE-VA2
32	[B] 6,7-dihydroxyflavanon	19.47	tr	-	-	-	-	-	-	-	-	-
33	Unidentified	19.52	-	-	-	tr	-	-	-	-	-	-
34	Unidentified	20.77	tr	-	-	-	-	-	-	-	-	-
35	[B] Apigenin-7-O-glucoside (Apigetrin)	20.97	-	tr	tr	tr	tr	tr	tr	-	-	-
36	Unidentified	21.21	-	-	-	-	tr	-	tr	tr	tr	-
37	Unidentified	21.46	-	tr	tr	tr	tr	tr	-	-	-	-
38	[B] Caffeic ethyl ester	22.45	-	-	-	-	tr	tr	-	tr	+	Tr
39	[B] Liquiritigenin	22.95	tr	-	-	-	-	-	-	-	-	-
40	[C] Aromadendrin (Dihydrokaempferol)	23.13	-	-	-	-	tr	tr	tr	-	-	-
41	[B] Pinobanksin-5-methylether	23.19	tr	++	+	++	++	++	+	+	tr	Tr
42	Unidentified	23.65	tr	-	-	-	-	-	-	-	-	-
43	Unidentified	23.81	+	-	-	-	-	-	-	-	-	-
44	[D] Caffeic acid derivate	23.87	-	tr	-	-	tr	-	tr	-	-	-
45	[B] 2'-Hydroxyformononetin	23.87	+	-	-	-	-	-	-	-	-	-
46	Unidentified	24.32	-	tr	-	-	-	-	-	-	-	-
47	[B] Odoratin	24.83	tr	-	-	-	-	-	-	-	-	-
48	[A] Quercetin-	24.93	-	tr	tr	tr	tr	tr	tr	tr	tr	-
49	[A] Luteolin	25.29	-	tr	tr	tr	tr	tr	tr	tr	tr	-
50	Unidentified	25.32	tr	-	-	-	-	-	-	-	-	-

No.	Component	RT	NE-TRI	GR-PA	KA-BO	UK-TA	GE-OR	GE-OT	GE-Q1	GE-Q2	GE-VA1	GE-VA2
51	Unidentified	25.44	tr	-	-	-	-	-	-	-	-	-
52	Unidentified	26.58	tr	-	-	-	-	-	-	-	-	-
53	[B] Quercetin-3-methyl ether	26.70	-	+	+	+	+	+	+	tr	tr	Tr
54	[A] Pinobanksin	26.99	tr	++	+++	++	+++	+++	++	++	+	+
55	Unidentified	27.97	+	-	-	-	-	-	-	-	-	-
56	[A] Naringenin	28.40	-	tr	tr	tr	tr	tr	tr	tr	tr	Tr
57	[B] Chrysin-5-methyl-ether	28.78	-	tr	tr	tr	-	tr	-	-	-	-
58	Unidentified	28.89	-	-	-	-	-	-	-	-	-	-
59	Unidentified	29.35	-	tr	tr	tr	tr	tr	tr	-	-	-
60	[C] Naringenin chalcone	30.09	-	tr	-	tr	-	-	-	-	-	-
61	[C] Aromadendrin-7-methyl ether	30.13	-	tr	tr	-	-	-	-	-	-	Tr
62	[A] Apigenin	30.41	-	+	+	+	+	+	+	+	+	Tr
63	[A] Kaempferol	31.01	-	tr	+	+	+	+	+	+	tr	Tr
64	[B] Caffeic acid hydroxyphenylethyl ester	31.26	-	tr	tr	tr	tr	tr	tr	tr	-	-
65	[A] Quercetin-3’-methyl ether (Isorhamnetin)	31.58	-	tr	tr	tr	tr	tr	tr	-	-	-
66	Unidentified	31.94	tr	-	-	-	-	-	-	-	-	-
67	[B] Quercetin-methyl-ether	32.12	-	tr	tr	tr	tr	tr	tr	-	-	-
68	[B] Luteolin-5-methyl ether	32.84	-	+	+	+	+	+	+	+	+	Tr
69	[B] Quercetin-di-methyl-ether isomer I	33.56	-	+	tr	tr	-	tr	tr	tr	tr	-

No.	Component	RT	NE-TRI	GR-PA	KA-BO	UK-TA	GE-OR	GE-OT	GE-Q1	GE-Q2	GE-VA1	GE-VA2
70	[B] Caffeic acid butenyl or isobutenyl ester	33.60	-	-	tr	-	tr	tr	tr	-	-	-
71	[B] Dalbergin	33.79	+	-	-	-	-	-	-	-	-	-
72	Unidentified	34.04	tr	-	-	-	-	-	-	-	-	-
73	[B] 1,3-di-*p*-Coumaroylglycerol	33.80	-	-	tr	tr	-	-	-	-	-	-
74	[B] Galangin-5-methyl-ether	34.27	-	+	tr	tr	tr	tr	tr	tr	-	-
75	[B] Pinobanksin-5-Methyl-ether-3-acetate	34.35	-	tr	tr	tr	tr	tr	tr	tr	-	-
76	[B] 7-Hydroxyflavanone	34.66	+	-	-	-	-	-	-	-	-	-
77	[B] 2-Acetyl-1,3-di-caffeoylglycerol	34.99	-	-	-	tr	-	-	tr	-	-	-
78	Unidentified	35.25	tr	-	-	-	-	-	-	-	-	-
79	[B] Isoliquiritigenin	35.38	tr	-	-	-	-	-	-	-	-	-
80	[A] Quercetin-7-methyl ether (Rhamnetin)	36.29	-	tr	tr	tr	tr	tr	tr	tr	tr	-
81	[B] Kaempferol-methyl-ether	36.44	-	tr	tr	tr	tr	tr	tr	tr	tr	-
82	[B] Caffeic acid butyl or isobutyl ester isomer I	36.91	-	tr	tr	-	tr	tr	tr	tr	tr	-
83	[B] Pinobanksin-3-hydroxybutyrate or isobutyrate	37.24	-	-	tr	tr	tr	tr	-		-	-
84	[B] Caffeic acid prenyl ester isomer	37.41	-	-	tr	-	+	tr	+	+	+	Tr
85	[B] Caffeic acid butyl or isobutyl ester isomer II	38.15	-	-	-	-	-	tr	-	-	-	-
86	[B] Quercetin--di-methyl-ether isomer II	38.55	-	+	tr	tr	+	+	tr	tr	tr	Tr
87	[B] Caffeic acid 2-methyl-2-butenyl ester	38.74	-	++	+++	++	+++++	+++	++++++	++++	+++	+
88	[B] Medicarpin	39.09	tr	-	-	-	-	-	-	-	-	-

No.	Component	RT	NE-TRI	GR-PA	KA-BO	UK-TA	GE-OR	GE-OT	GE-Q1	GE-Q2	GE-VA1	GE-VA2
89	[B] Caffeic acid 3-methyl-2-butenyl ester (Basic prenyl ester)	40.14	-	++	+++	++	++++++	++++	++++++	++++++	+++	+
90	[B] Caffeic acid 3-methyl-3-butenyl ester (Prenyl ester isomer I)	40.60	-	++	++	+	++	+	++	+++	+	Tr
91	[B] 2-Acetyl-1-caffeoyl-3-p-coumaroylglycerol	41.48	-	-	-	tr	-	-	-	-	-	-
92	[C] Methylated flavonoid	41.51	tr	-	-	-	-	-	-	-	-	-
93	[A] Chrysin	41.80	-	+++	+++	++++	+++++	++++	++++	+++	++	+
94	[B] Caffeic acid benzyl ester	41.95	-	++	+	++	+++	+++	+++	++	+	Tr
95	[A] Pinocembrin	42.64	+	++	+++	++++	+++++	++++	++++	+++	+	+
96	[A] Pinocembrin chalcone	42.90	-	++	-	-	-	-	-	-	-	-
97	[A] Naringenin 4’-methyl ether (Isosakuranetin)	43.77	-	+	+	+	+	+	+	tr	tr	Tr
98	[B] Ermanin isomer	43.98	-	-	tr	tr	tr	tr	tr	+	tr	-
99	[A] Galangina	44.42	-	++	+++	+++	++++	++++	++	++	+	+
100	[B] 4-Metoxydalbergione	44.91	tr	-	-	-	-	-	-	-	-	-
101	[A] Pinocembrin dihydrochalcone	45.01	-	tr	tr	-	tr	tr	-	-	-	-
102	[A] Apigenin-3’-methyl ether (Acacetin) or [A] Apigenin-7-methyl ether (Genkwanin)	45.14	-	tr	tr	tr	+	tr	+	+	+	-
103	[B] Caffeic acid pentyl or isopentylester	46.00	-	tr	tr	-	tr	tr	tr	-	tr	Tr
104	[A] Caffeic acid phenethyl ester (CAPE)	46.30	-	++	++	++	+++	++	++	+++	+	Tr
105	[B] Pinobanksin-3-acetate	46.40		+++	++++	+++++	++++++	++++++	++++	+++	++	+

No.	Component	RT	NE-TRI	GR-PA	KA-BO	UK-TA	GE-OR	GE-OT	GE-Q1	GE-Q2	GE-VA1	GE-VA2
106	A Kaempferide (Kaempferol 4'-methyl ether)	46.80	-	-	tr	-	tr	tr	tr	tr	-	-
107	B Methoxychrysin	47.23	-	+	tr	+	+	+	+	+	tr	Tr
108	B Ermanin (Kaempferol-3,4’-dimethyeter kemferolu)	49.46	-	tr	tr	tr	tr	tr	tr	tr	tr	-
109	B *p*-Coumaric acid 3-methyl-3-butenyl ester	49.69	-	-	tr	tr	tr	tr	tr	tr	tr	-
110	B 2-Acetyl-1,3-di-*p*-coumaroylglycerol	50.39	-	-	tr	+	-	-	-	-	-	-
111	B 2-Acetyl-3-*p*-coumaroyl-1-feruloylglycerol	51.06	-	-	-	tr	-	-	-	-	-	-
112	B *p*-Coumaric acid 3-methyl-2-butenyl or 2-methyl-2-butenyl	51.18	-	tr	tr	tr	+	tr	tr	tr	tr	-
113	Unidentified	51.35	tr	-	-	-	-	-	-	-	-	-
114	B 1-Acetyl-2,3-di-*p*-coumaroylglycerol	51.67	-	-	-	tr	-	-	-	-	-	-
115	B 2-Acetyl-1,3-di-feruloylglycerol	51.90	-	-	-	tr	-	-	-	-	-	-
116	Unidentified	52.52	-	tr	tr	tr	tr	tr	tr	-	-	-
117	B *p*-Coumaric acid benzyl ester	52.93	-	tr	tr	++	+	+	tr	+	tr	-
118	Unidentified	53.54	tr	-	-	-	-	-	-	-	-	-
119	Unidentified	54.27	-	+	tr	-	tr	tr	+	+	-	-
120	B Ferulic acid benzyl ester	54.53	-	-	-	tr	tr	tr	tr	-	-	-
121	B Caffeic acid cinnamyl ester	55.23	-	+	tr	+	++	++	+	+	tr	tr
122	B Pinobanksin-3-propanoate	57.35	-	++	+	+	+	+	tr	tr	tr	tr
123	B Caffeic acid hexyl or isohexyl ester	57.50	-	-	-	-	tr	-	tr	-	-	-

No.	Component	RT	NE-TRI	GR-PA	KA-BO	UK-TA	GE-OR	GE-OT	GE-Q1	GE-Q2	GE-VA1	GE-VA2
124	[B] *p*-Coumaric acid phenethyl ester	57.64	-	tr	tr	tr	tr	tr	tr	tr	-	-
125	[B] Pinostrobin chalcone	59.79	-	+	tr	tr	tr	+	tr	-	-	-
126	[C] 2’,6’-Dihydroxy–4’,4-dimethoxy dihydrochalcone (Calomelanone)	61.46	-	tr	tr	-	tr	tr	-	-	-	-
127	[A] Tectochrysin (Chrysin-7-methyl ether) [M+H]!	61.90	-	+	+	+	+	+	+	++	tr	tr
128	[A] Pinostrobin (Pinocembrin-7-methyl ether) [M+H]!	62.40	-	++	+	+	+	+	+	+	tr	-
129	[B] Pinobanksin-3-butenoate or isobutenoate	62.60	-	-	-	-	tr	tr	-	-	-	-
130	[B] *p*-Coumaric acid cinnamyl ester	63.68	-	tr	+	tr	+	+	tr	tr	tr	-
131	[B] Pinobanksin-3-butanoate or isobutanoate	64.47	-	++	++	++	++	++	+	+	tr	tr
132	Unidentified	64.88	-	-	-	tr	tr	tr	-	-	-	-
133	[B] Pinobanksin-3-pentenoate or isopentenoate I	65.16	-	+	+	+	+	+	tr	tr	-	tr
134	[B] Pinobanksin-3-pentenoate or isopentenoate II	65.48	-	tr	tr	tr	tr	tr	tr	tr	-	-
135	Unidentified	66.09	-	+	tr	tr	tr	tr	tr	tr	-	-
136	[D] Caffeic acid derivate	66.15	-	+	tr	-	tr	tr	tr	tr	-	-
137	[B] Pinobanksin-3-benzoate	66.61	-	-	tr	tr	tr	tr	-	-	-	-
138	[B] Pinobanksin-derivate	67.40	-	-	tr	-	-	-	-	-	-	-
139	[B] Pinobanksin-3-pentanoate or isopentenoate I	67.56	-	tr	+	+	tr	tr	tr	+	-	-
140	[B] Pinobanksin-3-pentanoate or isopentenoate II	67.70	-	+++	++	+++	++	++	+	+	tr	-
141	[C] Caffeic acid monoterpene (geranyl) ester	67.88	-	tr	+	-	+	+	+	-	-	-

No.	Component	RT	NE-TRI	GR-PA	KA-BO	UK-TA	GE-OR	GE-OT	GE-Q1	GE-Q2	GE-VA1	GE-VA2
142	Unidentified	67.98	-	-	+	+	+	+	-	-	-	-
143	[B] Pinobanksin-3-hexenoate or isohexenoate	68.36	-	-	tr	-	tr	tr	tr	tr	-	-
144	[B] Pinobanksin-3-hydroxycinnamate	69.05	-	tr	+	+	++	++	+	+	tr	tr
145	[B] Metoxycinnamic acid cinnamyl ester	69.11	-	+	+	+	++	++	+	tr	-	-
146	Unidentified	69.39	-	-	-	-	-	-	-	-	tr	+++
147	[B] Pinobanksin-3-hexanoate or isohexanoate I	69.40	-	tr	tr	tr	tr	tr	-	tr	-	-
148	Unidentified	69.56	-	-	-	-	-	-	-	-	+	+++
149	Unidentified	69.60	-	-	+	-	-	-	-	-	-	-
150	[B] Metoxycinnamic acid cinnamyl ester isomer	69.66	-	tr	tr	-	tr	tr	tr	tr	-	-
151	[B] Pinobanksin-3-hexanoate or isohexanoate II	69.70	-	tr	+	-	tr	tr	tr	tr	-	-
152	Unidentified	70.10	-	-	+	+	tr	tr	tr	tr	-	-
153	Unidentified	70.65	-	-	tr	-	tr	tr	tr	-	-	-
154	Pinobanksin-3-phenylpentenoate or phenylisopentenoate ester	70.73	-	-	tr	tr	+	+	tr	-	-	-
155	Unidentified	71.08	-	-	-	-	-	-	-	-	tr	++
156	Unidentified	71.19	-	-	tr	+	+	+	tr	-	-	-
157	Unidentified	71.29	-	-	-	-	-	-	-	-	tr	+++
158	Unidentified	71.37	-	-	tr	-	-	-	-	-	-	-
159	Unidentified	71.73	-	-	+	+	+	+	tr	tr	-	-

No.	Component	RT	NE-TRI	GR-PA	KA-BO	UK-TA	GE-OR	GE-OT	GE-Q1	GE-Q2	GE-VA1	GE-VA2
160	Unidentified	73.66	-	-	+	tr	-	-	-	-	-	-
161	Unidentified	76.54	-	-	-	-	+	-	++	++	+	+
162	Unidentified	76.75	-	-	-	-	+	-	+	+	+	tr

Propolis extracts acronyms: NE-TRI – Nepal, Trigona; GR-PA – Greece, Parga; KA-BO – Kazakhstan, Bozovoe; UK-TA – Ukraine, Tarnopol; GE-OR – Georgia, Orgora; GE-OT – Georgia, Ota; GE-Q1 – Georgia, Qvakhvreli sample 1; GE-Q2 – Georgia, Qvakhvreli sample 2; GE-VA1 – Georgia, Vasisubani sample 1; GE-VA2 – Georgia, Vasisubani sample 2.

Table Legend – Relative abundance units symbols used in the table:

MS evaluated components	Table symbol	Only tectochrysin and pinostrobin (UV units)	Table symbol
component not detected	-	component not detected	-
tr - intensity <5*10^4	tr	tr - height <1 mAU	tr
5*10^4< intensity >1.5*10^5	+	1< height >5 mAU	+
1.5*10^5< intensity >3*10^5	++	5< height >10 mAU	++
3*10^5< intensity >4.5*10^5	+++	10< height >15 mAU	+++
4.5*10^5< intensity>6.0*10^5	++++	15< height >20 mAU	++++
6.0*10^5< intensity>7.5*10^5	+++++	20< height >25 mAU	+++++
7.5*10^5< intensity>1.0*10^6	++++++	25< height mAU	++++++
1.0*10^6 < intensity	+++++++		

Table S3. Data input for correlation tests of general composition (TP, TF) and activities (DPPH, FRAP, IC50).

Propolis	Cytotoxicy tests			Antioxidant tests		General composition	
	**BJ** IC_{50} [µg/mL]	**A375** IC_{50} [µg/mL]	**SK-MEL-3** IC_{50} [µg/mL]	**DPPH** [mgGAE/g extract]	**FRAP** [mmolFe^{2+}/g extract]	**TF** [mgQUE/g extract]	**TP** [mgGAE/g extract]
**NE-TRI**	47.1	81.3	42.6	41.06	6.82	34.79	116.21
**GR-PA**	157.5	203.6	138.6	67.27	12.72	219.69	218.72
**KA-BO**	166.6	201.4	131.5	44.59	11.8	119.34	184.95
**UK-TA**	112	262.7	173.9	64.45	13.92	191.14	168.35
**GE-OT**	132.2	188.1	145.3	117.71	13.76	96.07	193.06
**GE-OR**	174.8	146.6	130.1	71.26	13.5	122.07	242.71
**GE-Q1**	121.2	124.1	147.8	116.38	16.83	81.5	218.7
**GE-Q2**	162.5	114.3	101.2	111.06	19.09	136.15	286.7
**GE-VA1**	183	59.9	138.6	88.71	13.75	106.8	227.55
**GE-VA2**	172.4	154.8	175.6	20.94	9.42	56.08	102.18

Table legend: units acronyms: mgGAE/g extract – mg of gallic acid equivalents per gram of dry extract; mmolFe^{2+}/g extract – mmol of Fe^{2+} equivalents per gram of dry extract; mgQUE/g extract – mg of Quercetin equivalents per gram of dry extracts. **Propolis extracts acronyms**: NE-TRI – Nepal, Trigona; GR-PA – Greece, Parga; KA-BO – Kazakhstan, Bozovoe; UK-TA – Ukraine, Tarnopol; GE-OR – Georgia, Orgora; GE-OT – Georgia, Ota; GE-Q1 – Georgia, Qvakhvreli sample 1; GE-Q2 – Georgia, Qvakhvreli sample 2; GE-VA1 – Georgia, Vasisubani sample 1; GE-VA2 – Georgia, Vasisubani sample 2. **Cell lines and tests acronyms:** BJ – normal human fibroblasts cell line; A375 – primary malignant melanoma cell line; SK-MEL – highly invasive melanomas originated from lymph cell line; DPPH – 2,2-diphenyl-1-picrylhydrazyl radical scavering activity; FRAP – ferric reducing antioxidant power assay; TF – flavonoid content; TP – total polyphenols content.

Table S4. Data input for correlation test of general composition (TP, TF) and caspase activity.

Propolis	Caspase activity levels			General composition	
	**BJ** [FI]	**SK-MEL-3** [FI]	**A375** [FI]	**TF** [mgQUE/g extract]	**TP** [mgGAE/g extract]
**GE-OR**	10020	15092	43759	136.15	286.7
**GE-VA1**	14526	11112	55864	106.8	227.55
**GE-Q2**	14498	8339	43241	56.08	102.18

Table legend: units acronyms: FI – fluorescence intensity mgGAE/g extract – mg of gallic acid equivalents per gram of dry extract; mgQUE/g extract – mg of Quercetin equivalents per gram of dry extracts. **Propolis extracts acronyms**: GE-OR – Georgia, Orgora; GE-Q2 – Georgia, Qvakhvreli sample 2; GE-VA1 – Georgia, Vasisubani sample 1. **Cell lines and tests acronyms:** BJ – normal human fibroblasts cell line; A375 – primary malignant melanoma cell line; SK-MEL – highly invasive melanomas originated from lymph cell line; DPPH – 2,2-diphenyl-1-picrylhydrazyl radical scavering activity; FRAP – ferric reducing antioxidant power assay; TF – flavonoid content; TP – total polyphenols content.
